# Multi-Site N-glycan mapping study 1: Capillary electrophoresis – laser induced fluorescence

**DOI:** 10.1080/19420862.2015.1107687

**Published:** 2015-10-14

**Authors:** Ákos Szekrényes, SungAe Suhr Park, Marcia Santos, Clarence Lew, Aled Jones, Ted Haxo, Michael Kimzey, Shiva Pourkaveh, Zoltán Szabó, Zoran Sosic, Peng Feng, Csaba Váradi, François de l'Escaille, Jean-Bernard Falmagne, Preeti Sejwal, Thomas Niedringhaus, David Michels, Gordon Freckleton, Melissa Hamm, Anastasiya Manuilov, Melissa Schwartz, Jiann-Kae Luo, Jonathan van Dyck, Pui-King Leung, Marcell Olajos, Yingmei Gu, Kai Gao, Wenbo Wang, Jo Wegstein, Samnang Tep, András Guttman

**Affiliations:** 1 Horváth Laboratory of Bioseparation Sciences; University of Debrecen; Debrecen, H-4032, Hungary; 2 Drug Product Development; P&PD; Amgen; Inc.; Thousand Oaks, CA 91320, USA; 3 SCIEX; Brea, CA 92821, USA; 4 ProZyme; Inc.; Hayward, CA 94545, USA; 5 Analytical Development; Biogen; Cambridge, MA 02142, USA; 6 Analis s.a. R&D Diag.; Suarlée (Namur), Belgium; 7 Bioanalytical and Discovery Analytical Sciences; Bristol-Myers Squibb; Lawrenceville, NJ 08648, USA; 8 Protein Analytical Chemistry Department; Genentech; Inc.; South San Francisco, CA 94080, USA; 9 Bioanalytical Sciences; Eli Lilly and Company (previously ImClone); Branchburg, NJ 08876, USA; 10 Vaccine Bioprocess Research and Development; Merck Research Laboratories; West Point, PA 19486, USA; 11 Analytical Research and Development; Pfizer; Inc.; Andover, MA 01810, USA; 12 Analytical Science; Boehringer Ingelheim; Inc.; Fremont, CA 94555, USA; 13 Regeneron Pharmaceuticals; Inc.; Tarrytown, NY 10591, USA; 14 Analytical Sciences; Seattle Genetics; Inc.; Bothell, WA 98021, USA; 15 ImmunoGen; Inc.; Waltham, MA 02451, USA; 16 Analytical Research and Development; Gedeon Richter; Plc.; Budapest, H-1475, Hungary; 17 Eli Lilly and Company; Indianapolis; IN 46285, USA; 18 Division of Monoclonal Antibody; National Institutes for Food and Drug Control; Beijing, PR China; 19 MTA-PE Translational Glycomics Group; MUKKI; University of Pannonia; Veszprém, Hungary; † Current affiliation: Process Analytical, AbbVie, North Chicago, IL 60064, USA.

**Keywords:** Biotherapeutics, Capillary electrophoresis, Intercompany study, N-glycans

## Abstract

An international team that included 20 independent laboratories from biopharmaceutical companies, universities, analytical contract laboratories and national authorities in the United States, Europe and Asia was formed to evaluate the reproducibility of sample preparation and analysis of N-glycans using capillary electrophoresis of 8-aminopyrene-1,3,6-trisulfonic acid (APTS)-labeled glycans with laser induced fluorescence (CE-LIF) detection (16 sites) and ultra high-performance liquid chromatography (UHPLC, 12 sites; results to be reported in a subsequent publication). All participants used the same lot of chemicals, samples, reagents, and columns/capillaries to run their assays. Migration time, peak area and peak area percent values were determined for all peaks with >0.1% peak area. Our results demonstrated low variability and high reproducibility, both, within any given site as well across all sites, which indicates that a standard N-glycan analysis platform appropriate for general use (clone selection, process development, lot release, etc.) within the industry can be established.

## Abbreviations

ANOVA: Analysis of Variance; APTS, 8-aminopyrene-1,3,6 trisulfonic-acid
CE-LIF: Capillary Electrophoresis – Laser Induced Fluorescence
CE-SDS: Capillary Sodium Dodecylsulfate gel electrophoresis
GU: Glucose Unit
UHPLC: Ultra High-Performance Liquid Chromatography

## Introduction

Recent rapid expansion in the field of biopharmaceutical product development and the concomitant commercialization of therapeutic proteins has increased the need to implement rapid and reproducible analytical methods for monitoring important post-translational modifications of the product, such as N-glycosylation. Regulatory agencies consider N-glycosylation to be a critical quality attribute due to the potential effects on pharmacokinetics, biological activity, stability and immunogenicity.^1^ Product quality attribute evaluation in the biopharmaceutical industry often requires the use of complementary analytical methodologies (e.g., capillary electrophoresis, liquid chromatography) to alleviate the risks associated with using only one analytical tool.^2^ Additionally, applying orthogonal separation techniques can help provide more consistent and more thorough characterization of lots. In the past few years, capillary electrophoresis with laser-induced fluorescent detection (CE-LIF) has become an important bioanalytical tool in the biotechnology and biopharmaceutical industries to provide comprehensive glycosylation analysis data. 3 In CE-LIF-based glycan analysis, neutrally coated capillaries are used for the separation of fluorophore labeled (8-aminopyrene-trisulfonic acid, APTS) carbohydrate molecules. CE provides a directly orthogonal approach to liquid chromatography and mass spectrometry, and therefore its use addresses requests from regulatory agencies for orthogonality in product characterization.

During the various steps of the development process, CE can provide identification and relative quantitation of glycans in a rapid or high throughput manner with high sensitivity. This allows fast turnaround times for glycan analysis and the ability to monitor product consistency, as well as potentially immunogenic glycan epitopes. There are, however, some limitations, as co-migration of some glycans may occur. In the method presented here, one glycan of interest (Man5) is observed to co-migrate with another glycan. This can be addressed using the same approach as one would use in chromatography; exoglycosidase treatment to remove the co-migrating species would allow quantitation of the Man5 glycan. Additionally, the use of various exoglycosidases can help provide the elucidation of other glycan structures. Glycan analysis by CE can also be utilized in a GMP environment to provide results for comparability analysis and product release.

To prove the reproducibility and transferability across sites of CE-LIF as a routine analytical methodology for N-glycan analysis of biopharmaceuticals, an intercompany collaboration^3-5^ (initially formed at the CASSS CE Pharm 2012 meeting) was formed with 16 laboratories located in North America, Europe and Asia from independent industrial and academic participants. This endeavor assessed intra- and inter-laboratory repeatability and reproducibility (different laboratories, different personnel, multiple instruments and different days of analysis) of the selected sample preparation^6^ and CE-LIF method used. A labeled glycan standard ready for analysis and a glycoprotein were chosen as test articles to assess the contribution to variability of the analysis alone *vs*. that of the sample preparation and analysis. After a predefined system suitability test to assure that systems were performing optimally, the laboratories analyzed 6 different samples including a pre-labeled homo-oligomer ladder standard, 3 pre-labeled glycan standards (containing only high mannose and complex type afucosylated and fucosylated glycans), released and pre-labeled glycan test article sample and 3 parallel-prepared glycoprotein test articles. Statistical analysis of the obtained results was performed following ISO 5725–2 guideline principles.^7^ Glucose Unit (GU) values were determined^2,8^ for all peaks and the data provided by each laboratory was critically evaluated to identify outliers.

The goal of this interlaboratory study was, after agreement among the participants, to use the most advanced procedures and methods currently available to evaluate comparability of results across sites in order to expedite regulatory and industrial recognition of CE-LIF methods as a reliable toolset to comprehensively characterize the N-glycosylation patterns of therapeutic proteins. To this end, calibration procedures and best practices, detailed protocols and a 2-hour training webinar were provided by the respective suppliers to ensure that participants understood the procedures. The results of the system suitability injections were evaluated to assure that all instruments were calibrated and running properly.

## Results

**Figure 1.**
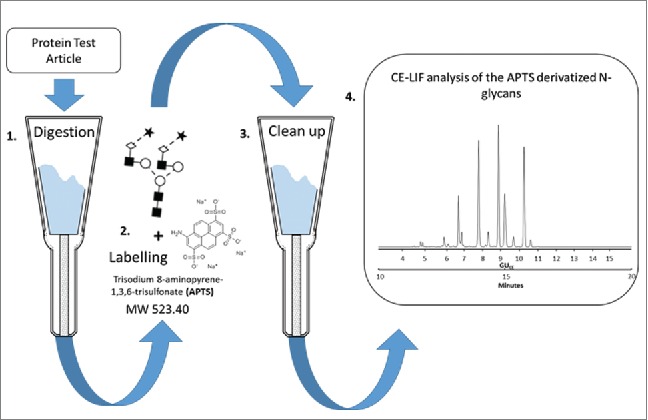
Schematic representation of the overall strategy for N-glycan analysis of the Protein Test Article using CE-LIF. 1) Peptide *N*-glycosidase F-mediated release of N-linked carbohydrates was followed by 2) a rapid reductive amination-based labeling reaction with APTS. 3) In the next step, salts and unbound dye molecules are removed from the sample and the APTS derivatized glycans are eluted by HPLC-grade water. 4) Finally, the samples are analyzed by CE-LIF.

A schematic representation of the sample preparation workflow is shown in **Figure 1**. The samples were first denatured and loaded onto the cartridges for digestion. The released glycans were then isolated, dried and labeled with APTS via reductive amination. The labeled glycans were cleaned up from excess labeling reagents and analyzed by CE-LIF. **Table 1** provides data for inter-laboratory reproducibility for relative peak areas, including all participating laboratories. The means for each peak from each site are presented along with the overall means. The %RSD represent the overall inter-laboratory reproducibility and is displayed as a heatmap, with green being low and red being high.

**Table 1.** Inter-laboratory reproducibility for relative peak areas, including all participating laboratories. An elevated %RSD is observed for some peaks due to the inclusion of results from outlying sites.

		Mean of replicate injections by site																	
Peak #	Name (UOXF)	A	B	D	E	F	G	H	I	J	K	L	M	N	O	P	S	Overall Mean	%RSD
1	A2G2S2	0.07	0.13	0.11	0.13	0.12	0.11	0.14	0.12	0.10	0.12	0.12	0.12	0.12	0.12	0.13	0.13	0.12	13.68%
2	FA2G2S2	0.64	0.76	0.71	0.77	0.71	0.73	0.74	0.72	0.70	0.76	0.74	0.72	0.79	0.72	0.77	0.77	0.73	5.08%
3	FA2BG2S2	0.55	0.63	0.57	0.63	0.62	0.61	0.61	0.60	0.61	0.62	0.62	0.59	0.66	0.60	0.64	0.65	0.61	4.81%
4	FA2[6]G1S1	0.20	0.21	0.20	0.21	0.22	0.22	0.21	0.22	0.21	0.21	0.24	0.20	0.22	0.22	0.21	0.20	0.21	4.56%
5	FA2[3]G1S1	1.69	1.68	1.65	1.70	1.69	1.69	1.70	1.65	1.68	1.70	1.66	1.68	1.71	1.65	1.69	1.68	1.68	1.11%
6	A2G2S1	0.39	0.52	0.49	0.49	0.48	0.50	0.53	0.52	0.47	0.52	0.50	0.57	0.50	0.52	0.50	0.57	0.51	8.14%
7	A1	0.12	0.18	0.16	0.14	0.15	0.18	0.18	0.18	0.15	0.18	0.17	0.17	0.18	0.18	0.17	0.22	0.17	13.34%
8	FA2G2S1+A2	8.09	8.67	8.39	8.69	8.37	8.69	8.69	8.47	8.49	8.67	8.68	8.70	8.70	8.46	8.62	8.63	8.56	2.01%
9	FA2BG2S1+M5	2.78	2.47	2.42	2.42	2.61	2.38	2.41	2.54	2.28	2.45	2.47	2.59	2.53	2.54	2.45	2.74	2.51	5.16%
10	Unknown 1	0.36	0.25	0.23	0.23	0.26	0.26	0.25	0.26	0.28	0.26	0.24	0.32	0.26	0.26	0.26	0.29	0.27	12.12%
11	A2B	0.09	0.15	0.12	0.12	0.11	0.14	0.13	0.16	0.10	0.14	0.38	0.12	0.12	0.16	0.12	0.14	0.15	46.29%
12	FA2+M6+A2[6]G1	21.01	20.77	21.26	20.93	20.80	20.74	20.74	20.75	20.83	20.82	20.52	20.64	20.69	20.74	20.74	20.53	20.78	0.86%
13	A2[3]G1	0.25	0.33	0.31	0.28	0.32	0.31	0.32	0.35	0.28	0.32	0.30	0.31	0.30	0.36	0.30	0.35	0.31	8.79%
14	FA2B	2.94	3.00	2.97	2.96	2.93	2.98	2.98	2.98	2.96	3.00	3.36	2.83	2.94	2.98	2.97	2.96	2.98	3.63%
15	FA2[6]G1+M7	23.05	22.73	23.02	23.00	22.86	22.88	22.81	22.91	23.01	22.81	22.55	22.48	22.74	22.90	22.89	22.41	22.82	0.84%
16	FA2[3]G1+A2G2+FA2B[6]G1	14.19	14.01	14.09	13.87	14.12	14.05	14.03	14.05	14.11	13.99	13.79	14.18	13.96	14.05	14.06	14.02	14.04	0.72%
17	M8+FA2B[3]G1	1.95	2.17	2.09	2.12	2.05	2.03	2.12	2.19	2.00	2.11	2.54	2.12	2.17	2.20	2.00	2.31	2.14	6.65%
18	FA2G2+M9	19.94	19.76	19.69	19.75	19.97	19.88	19.85	19.76	20.16	19.73	19.55	19.97	19.84	19.75	19.89	19.77	19.83	0.72%
19	FA2BG2	1.56	1.45	1.41	1.42	1.48	1.48	1.44	1.45	1.46	1.45	1.42	1.56	1.45	1.45	1.47	1.50	1.47	2.94%
20	Unknown 2	0.12	0.13	0.12	0.13	0.14	0.13	0.13	0.13	0.12	0.12	0.13	0.13	0.14	0.13	0.13	0.13	0.13	4.03%

**Figure 2.**
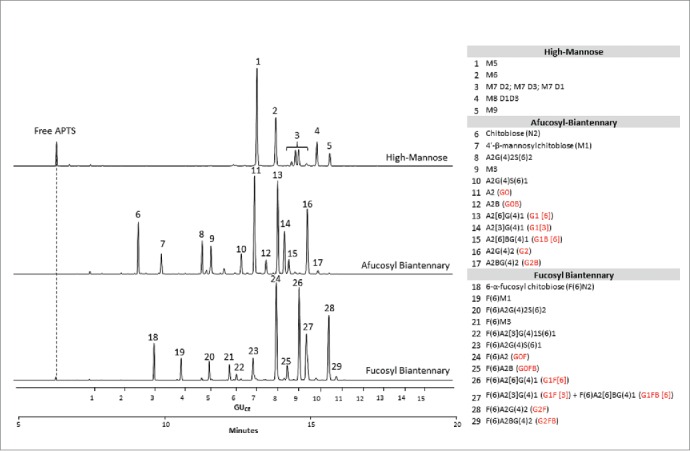
CE-LIF analysis of APTS-labeled partitioned N-glycan libraries of high-mannose (upper trace), fucosyl biantennary (lower trace) and afucosyl biantennary (middle trace) structures. All identified N-glycan structures are listed in the right panel with their Oxford notation.^9^ Commonly used names (highlighted by red in brackets) are also given for structures that can usually be found on therapeutic antibodies.

**Figure 2** depicts the CE-LIF traces of the APTS-labeled partitioned N-glycan libraries (APTS-labeled glycan libraries are commercially available products; see supplemental section for information). The upper trace shows the analysis of the high-mannose glycans featuring excellent separation of the positional mannose isomers of M7 and M8 structures. The middle trace displays the separation of afucosylated biantennary sugars. The lower trace shows the analysis of the fucosyl biantennary library; co-migration of G1F[3] and G1F[6]B is observed in peak 27. Based on the comparison of the 3 traces, one can predict that some of the peaks have the tendency to co-migrate when a full mixture of the glycan species represented by the partitioned libraries are present.

**Figure 3.**
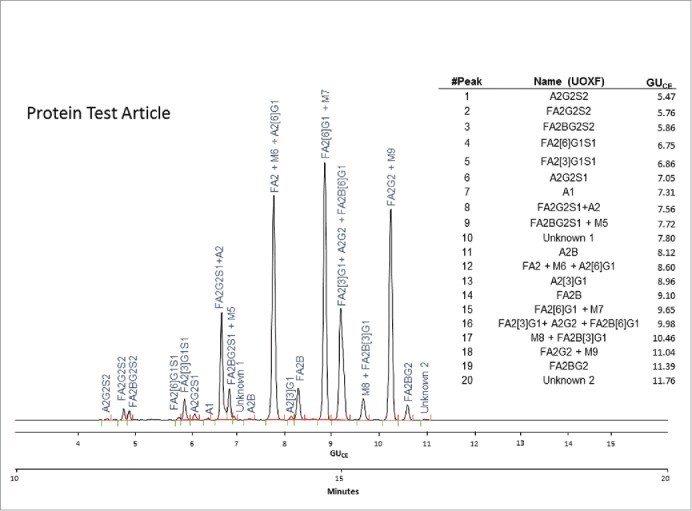
CE-LIF analysis trace of the APTS-labeled N-glycan profile of the Protein Test Article. The 20 most-abundant peaks were integrated in all the submitted profiles and their migration times and relative and total areas were then used to determine internal precision and reproducibility of the N-glycan mapping assay. The right panel shows the corresponding GU_CE_ values for all integrated peaks in their migration order, as well as the name of all identified structures using Oxford notation.^9^

**Figure 3** displays the CE-LIF N-glycan profile of the Protein Test Article showing 20 peaks from the mixture of 27 identified structures. The sample was spiked with 8% high-mannose-type glycans (M5-M9); therefore, some of those glycans co-migrated with certain complex structures and appear as either tailing or fronting peaks. This type of electrophoretic profile was consistently obtained by all laboratories.

## Quantitative Analysis of the Protein Test Article

All electropherograms were integrated by the study coordinators using the same integration parameters. Migration times, absolute, relative and total peak area values were determined for the 20 peaks found in the Protein Test Article N-glycan profiles (**Fig. 3**). Glycan structures were assigned based on the Partitioned N-Linked Glycan Libraries.

**Figure 4.**
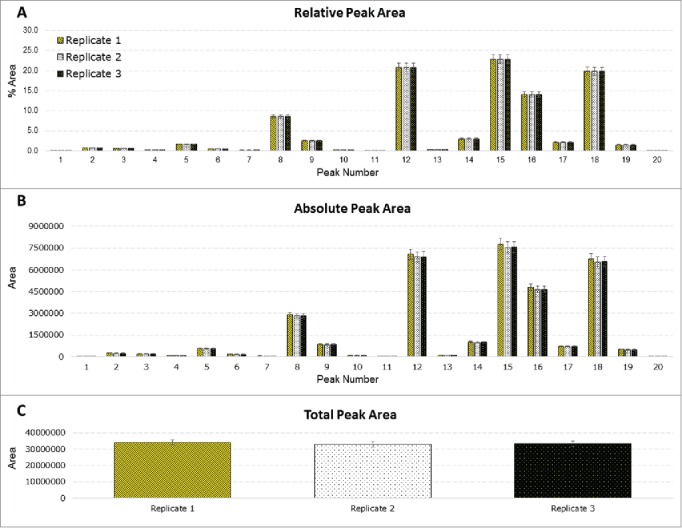
Statistical analysis of site F. Three chemical replicates were prepared; 3 injections were performed for each replicate. (A) Relative peak area for each integrated peak. (B) Absolute peak areas for each integrated peak. (C) Total peak area of each replicate.

Statistical analysis was performed to assess intra-laboratory repeatability for absolute, relative and total peak areas; this was performed for each of the participating laboratories. The results from a randomly selected site, site F, are presented in **Figure 4**. The results obtained at site F show very low variability in the results, highlighting the precision of the method. The corresponding results for the remaining sites are contained in the Supplemental Data section; most of the remaining sites showed similar results. Inter-laboratory reproducibility (for all participating laboratories) for relative peak areas was also assessed (**Table 1**). The results compiled from all the sites revealed some differences in quantitation at some sites, which resulted in an elevated %RSD for some peaks. Outlier analysis would confirm that some sites were of high variance from the mean.

The procedure described in the ISO 5725–2 for outlier removal was applied based on migration time and %Area, separately, for each of the 20 peaks across all sites. First, a visual evaluation for consistency of the results and laboratories was performed by using the Mandel's *k* and *h* statistics. The Cochran's and Grubb's tests were then applied to reject data in individual or outlier laboratories, respectively. There were 2 possible types of outliers: (1) outlying results for each of the peaks within the individual laboratories, meaning that the data was too low or too high compared to the other results; and (2) outlying laboratories that deviate either in precision or in mean values compared to the other laboratories. The statistic *k* measured the intra-laboratory consistency by comparing the standard deviation of the percent peak area values for a selected peak within one laboratory to the mean standard deviation of the different laboratories for that same peak. The homogeneity of the variances was then tested with the Cochran's test.

**Figure 5.**
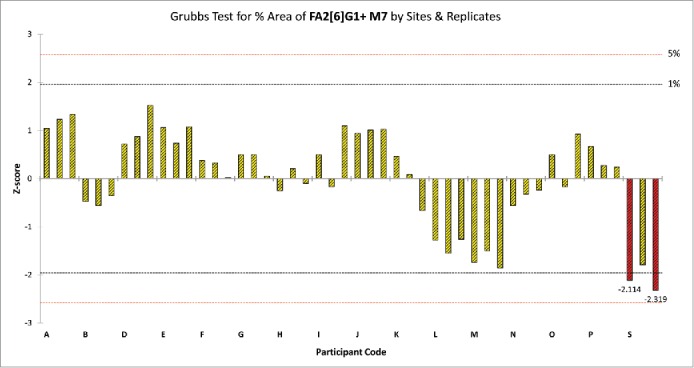
Grubb's test for %Area of peak 15 in the Protein Test Article by sites and replicates. Red columns represent those replicate samples of site S where the %Area value of peak 15 were considered.

**Figure 6.**
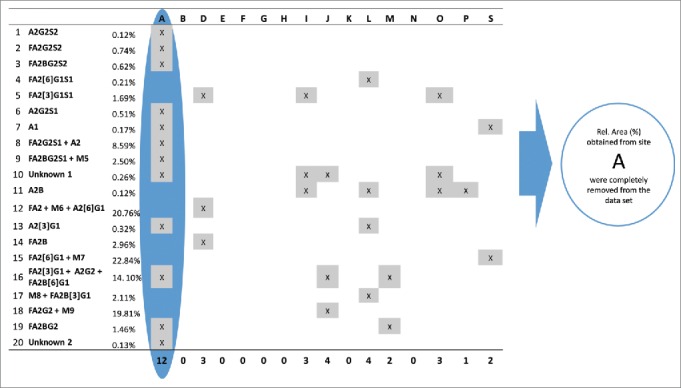
Identification of outlying relative %Area values for all sites and peaks. Peaks are listed in their migration order. If a site had more than 5 outlier values in its data set, the results obtained from that site were removed from the data set and were not used in further calculations. Since site A obtained outlying %Area values (highlighted by blue) for 12 peaks its data were rejected and were not used during the final calculation of assay repeatability and reproducibility.

As an example, **Figure 5** displays the results of the Grubb's test for the average %Area values of peak 15 by sites and replicates. In this case 2 outlying values (red columns) were identified and should be removed from the data set. **Figure 6** shows the result of outlier analysis for relative peak areas by sites and peaks. If any laboratory had more than 5 peaks in its Protein Test Article profiles, which were deviating either in precision or mean values compared to other laboratories, all of its data was eliminated from the data set and outlier analysis was repeated on the reduced data set. Since site A obtained deviating %Area values for more than half of the peaks in all Protein Test Article replicates, its data were not used in the calculation of the final results; migration time values obtained from site A were also outliers for most peaks. This indicated that either sample preparation or the separation conditions were different in this laboratory. A subsequent investigation found that the separation temperature used by site A was 25°C instead of 20°C, as defined in the study protocol. Due to the higher separation temperature, the viscosity of the separation buffer decreased, which resulted in shorter migration times. Decreased viscosity of the separation media affected the peak shapes, and affected resolution of the injected sample. Since most of the integration parameters were fixed during the data evaluation, this decrease in resolution and change in peak shape resulted in deviating %Area values for more than half of the integrated peaks in the profile.

After the outlier analysis was completed, the results obtained from 3 separate injections of the replicate runs were taken and migration time means were calculated for each peak of the replicates. All integrated peaks in the Protein Test Article profiles were then calibrated against the mean of 2 APTS-labeled maltodextrin ladder runs, and assigned glucose-unit values by fitting a fifth-order polynomial distribution curve to generate GU_CE_ values. Glucose-unit values were calculated from the migration-time means of certain peaks based on 3 parallel injections and from the migration-time means of the 2 glucose homo-oligomer ladder runs, which were injected prior to and after the actual Protein Test Article. Analyses of the pre-labeled ladder standard were very reproducible across the sites (**Table 2**). Both migration times and %Area of the ladder peaks (only data for the G2 peak presented) were generally very stable between injections; means were calculated from the 6 injections performed at each site.

**Table 2.** Reproducibility of the G2 (maltose) peak of the pre-labeled malto-oligomer ladder by sites. The table contains the mean values obtained from summarizing up 6 consecutive injections carried out by each site. No outlier analysis was performed on the data set before calculating the overall means. (USP: United States Pharmacopeial Convention)

Reproducibility of the G2 (maltose) peak by sites				
Site	Migration time	Theoretical Plates (USP)	Resolution (USP)	Area%
B	7.85	423551.67	30.47	1.50
D	7.46	452146.00	31.76	1.67
E	7.78	412878.00	29.90	1.60
F	7.59	369468.00	30.95	1.46
G	7.64	404761.00	29.77	1.51
H	7.94	417004.83	30.38	1.77
I	7.72	414227.17	29.41	1.56
J	7.95	426155.50	31.02	1.61
K	7.94	429485.17	30.65	1.60
L	7.85	423551.67	30.47	1.50
M	7.92	419358.50	30.42	1.33
N	7.84	419616.83	30.44	1.53
O	7.69	411039.17	29.62	1.55
P	7.63	411859.33	29.79	1.48
S	7.97	433776.33	31.08	1.68
*Mean*	*7.78*	*417925.28*	*30.41*	*1.56*
*SD*	*0.16*	*17601.80*	*0.64*	*0.11*
*%RSD*	*2.01*	*4.21*	*2.09*	*6.86*

Finally, repeatability, variation in the results of within a laboratory, and reproducibility, variation in the results between the laboratories, were determined for both migration times and %Peak area by peaks. **Table 3** summarizes the overall mean, the repeatability, and reproducibility of each peak. In **Table 3**, Panel A contains the calculated mean %Area values and their relative standard deviation means for all the 20 peaks larger than 0.1%; Panel A is divided into 3 parts. The upper part contains peaks with relative area between 0.1% and 1%. The mean repeatability for these low-abundance structures was 1.53%, while the mean reproducibility across the laboratories was 7.30%. The middle part contains peaks with relative area between 1% and 10%; the mean reproducibility across the laboratories for these peaks was 2.63%. The four major peaks (relative areas >10%) had a mean repeatability and reproducibility 0.30% and 0.80%, respectively (lower section of panel A). The general means of repeatability and reproducibility for %Area were 0.95% and 4.60%, respectively. Panel B in **Table 3** shows the overall mean of the migration times by peak. The mean repeatability and reproducibility of migration time were 0.06% and 2.24%, respectively. These results demonstrate that the study assay was robust and highly reproducible, which affirms its usefulness in the biopharmaceutical industry. Additionally, the observed reproducibility indicates the potential to validate the method presented for the relative quantitation of glycans. A 20% CV for peaks between 0.1 and 1.0% would be suggested as this would comply with industry guidelines and standards to ensure accurate analysis. Accurate glycan analysis can allow for the determination of relevant indices (e.g., fucosylation, galactosylation mannosylation, sialylation), which can be used to predict biological effects (e.g., pharmacokinetics/pharmacodynamics, antibody-dependent cell-mediated cytotoxicity, complement-mediated cytotoxicity).

**Table 3.** Summary of %Area, migration time repeatability and reproducibility values obtained from the Multi-Site N-Glycan study. **(A)** Summary of the overall mean %Area values for the 20 most-abundant peaks and their repeatability and reproducibility. **(B)** Summary of the calculated general mean migration times and their reproducibility and repeatability by peaks.

%Area	Peak #	Name (UOXF)	%Area	Repeatability (%RSD)	Reproducibility (%RSD)	Mean Reproducibility (%RSD)
						
<1%	1	A2G2S2	0.12	1.22%	8.63%	7.30%
	11	A2B	0.12	3.16%	15.46%	
	20	Unknown 2	0.13	2.16%	3.63%	
	7	A1	0.17	2.26%	9.80%	
	4	FA2[6]G1S1	0.21	1.43%	3.46%	
	10	Unknown 1	0.26	1.93%	9.45%	
	13	A2[3]G1	0.32	1.60%	7.22%	
	6	A2G2S1	0.51	0.41%	5.78%	
	3	FA2BG2S2	0.62	0.54%	4.95%	
	2	FA2G2S2	0.74	0.62%	4.58%	
<10%	19	FA2BG2	1.46	0.32%	2.37%	2.63%
	5	FA2[3]G1S1	1.69	0.29%	0.94%	
	17	M8 + FA2B[3]G1	2.11	0.51%	4.54%	
	9	FA2BG2S1+M5	2.50	0.71%	4.96%	
	14	FA2B	2.96	0.36%	1.44%	
	8	FA2G2S1+A2	8.59	0.28%	1.54%	
>10%	16	FA2[3]G1+A2G2+FA2B[6]G1	14.10	0.76%	1.12%	0.80%
	18	FA2G2+M9	19.81	0.16%	0.62%	
	12	FA2+M6+A2[6]G1	20.77	0.15%	0.68%	
	15	FA2[S]G1+M7	22.84	0.12%	0.77%	
*Mean*	*0.95%*	*4.60%*				

**B**						
**Peak #**	**Name (UOXF)**	**Mean Migration Time (min)**	**Repeatability (%RSD)**	**Reproducibility (%RSD)**	**Mean GU**_**CE**_	**Reproducibility for GU**_**CE**_ (%RSD)
1	A2G2S2	11.41	0.06%	2.41%	5.47	0.21%
2	FA2G2S2	11.67	0.06%	2.38%	5.76	0.18%
3	FA2BG2S2	11.76	0.05%	2.37%	5.86	0.18%
4	FA2[6]G1S1	12.52	0.05%	2.33%	6.75	0.14%
5	FA2[3]G1S1	12.61	0.06%	2.32%	6.86	0.13%
6	A2G2S1	12.77	0.05%	2.30%	7.05	0.13%
7	A1	12.98	0.06%	2.29%	7.31	0.12%
8	FA2G2S1+A2	13.18	0.06%	2.27%	7.56	0.12%
9	FA2BG2S1+M5	13.30	0.06%	2.32%	7.72	0.12%
10	Unknown 1	13.37	0.06%	2.24%	7.80	0.21%
11	A2B	13.61	0.06%	2.23%	8.12	0.09%
12	FA2+M6+A2[6]G1	13.97	0.06%	2.24%	8.60	0.10%
13	A2[3]G1	14.25	0.05%	2.21%	8.96	0.09%
14	FA2B	14.36	0.05%	2.20%	9.10	0.08%
15	FA2[S]G1+M7	14.77	0.06%	2.17%	9.65	0.09%
16	FA2[3]G1+A2G2+FA2B[6]G1	15.01	0.06%	2.15%	9.98	0.09%
17	M8 + FA2B[3]G1	15.36	0.06%	2.13%	10.46	0.09%
18	FA2G2+M9	15.79	0.06%	2.09%	11.04	0.12%
19	FA2BG2	16.05	0.06%	2.06%	11.39	0.12%
20	Unknown 2	16.32	0.06%	2.04%	11.76	0.14%
*Mean*			*0.06%*	*2.24%*		*0.13%*

## Discussion

Inter-laboratory studies fulfill several requirements of the quality management of bioanalytical measurements. They contribute to the validation of analytical methods, assess the proficiency of individual laboratories, estimate measurement uncertainty and certify test articles in a wide range of application and fields. The glycosylation profiles and quantitated values of the study's test articles were consistent for all organizations involved in the study. This study has shown that the applied CE-LIF method for the analysis of glycans provides reliable intra- and inter-laboratory results across laboratories in North America, Europe and Asia, and it is therefore a robust tool for N-glycan analysis that can confidently be applied on its own or as an orthogonal/complementary technique to other existing methods.

It is anticipated that this kit-based sample preparation combined with CE-LIF analysis will help to expedite pharmaceutical processes in all areas of the biopharmaceutical industry, and that the results presented in this study will highlight the applicability and utility of capillary electrophoresis as a bioanalytical tool.

## Materials and Methods

### Chemicals and reagents

Various reagents, consumables and all samples were provided by ProZyme Inc. (Hayward, CA, USA) and SCIEX (previously Beckman Coulter, Brea, CA, USA). All capillary columns, reagents, consumables and samples, which were sent to the participating laboratories, were from the same production batches. The Protein Test Article was a mixture of human IgG and a small amount of bovine RNase B (the glycan components were identified by mass spectrometry prior to distribution). A complete list of samples, reagents and other materials used (including product and lot numbers) can be found in the Supplemental Information section.

### Sample preparation

All glycan standards, glycan libraries and the glycan test article were provided to each site as pre-labeled, purified (i.e., dye free) dry aliquots; the Protein Test Article was provided to each site as individual samples from the same production batch. Each independent laboratory performed sample preparation using ProZyme's GlykoPrep Rapid N-Glycan Preparation for glycan release, derivatization with APTS and clean up. Protein Test Article samples were prepared in triplicate; the detailed sample preparation protocol is outlined in the Supplemental Information section. Standards and control samples were kept at −20 °C when not in use and were allowed to equilibrate to room temperature and vortexed prior to CE analysis.

### Capillary electrophoresis

Capillary electrophoresis analyses of the APTS-labeled N-glycans were performed in a PA 800 plus Pharmaceutical Analysis System (SCIEX Brea, CA) equipped with a fluorescence detector (excitation 488 nm, emission 520 nm, acquisition rate: 4 Hz). The separations were accomplished using 60 cm N-CHO coated capillaries (50-cm effective length; 50 μm i.d.) filled with the N-CHO Carbohydrate Separation Gel Buffer (SCIEX The capillaries were rinsed with the separation gel-buffer for 3 min at 30 psi before each run. The applied electric field strength was 500 V/cm, with the cathode at the injection side and the anode at the detection side (reversed polarity). All separations were accomplished at 20°C. Samples were stored at 10 °C and injected by pressure: 2 psi (13.8 kPa) for 10 sec. The software package, 32Karat version 9.0 (SCIEX was used for data acquisition and analysis).

### System certification test

As the first step, each laboratory had to perform a test analysis running the exact same test sequence including pre-labeled standards and glycan test article samples. After completing the analysis, the obtained data were submitted to the study coordinators for data evaluation. The system certification test guaranteed that laser powers and detector sensitivities were within a predefined range, all units were suitable for the analysis and also ensured that all obtained and submitted glycan profiles would be comparable during the study. Detailed descriptions of the CE system certification test, criteria and the injection sequence are listed in the Supplemental Information section.

### Study design

The goal of the study was to evaluate the variability of N-glycan analysis across multiple sites using a standardized CE-LIF method, adhering to our rigorous performance criteria. All peaks with ≥0 .1% peak area were evaluated; outlier analysis was performed using Grubb's and Cochran's C tests.

Study components (glycan and glycoprotein test articles and sample preparation kits provided by ProZyme; N-CHO capillaries, N-CHO separation buffer, conditioning and separation method provided by SCIEX were sent out to each of the laboratories. Samples were prepared in triplicate and each replicate was injected 3 times in a pre-defined sequence (see Supplemental Information section). The raw data files were then submitted to the study coordinators. Peak areas, relative peak areas and migration times were evaluated using the same integration parameters; GU_CE_ values were also determined for all peaks. After the data was evaluated, statistical and outlier analyses were performed (see paragraph below); outliers were removed from the data set. After removal of the outliers, intra- and inter-laboratory mean %RSD of migration times and %Area were determined for all peaks ≥0 .1%; intra-laboratory mean total signal was evaluated for each data set as well. Participant names were letter coded to “blind” the results; for cases in which more than one laboratory from a single organization participated, they were listed separately.

## Statistical Data Evaluation and Outlier Analysis

Statistical (repeatability and reproducibility) and outlier analyses were performed based on ISO 5725–2 guide^7^ principles. Intra-laboratory analysis (repeatability) was assessed for relative peak area, absolute peak area and total peak area for each site; inter-laboratory analysis (reproducibility) was assessed for relative peak area only because it is expected that absolute and total peak areas would likely vary widely due to differences related to the fluorescence detectors between sites. All of the data received from the participating laboratories were critically examined for outlier identification: i) outlying laboratories, which deviate in precision (repeatability) indicating laboratory bias; ii) outlying results from individual laboratories at a given level that deviate either in precision or in their mean value. From a statistical point of view, unequal repeatability values deviate from a fundamental assumption of analysis of variances (ANOVA), namely homogeneity of variances. Too low or too high values lead to distributions that are no longer normal. ISO recommends Mandel k and h statistics, respectively, obtained as follows:

(Eq. 1) $$k_{ij}=\frac{s_{ij}\;\sqrt{P_{j}}}{\sqrt{\sum_{i=1}^{P}s_{ij}^{2}}}$$

and

(Eq. 2) $$h_{ij}=\frac{{\bar{y}}_{ij}-{\bar{y}}_{j}}{\sqrt{\frac{1}{P_{j}-1}{\sum^{\text{​}}}_{i=1}^{P_{j}}{\left({\bar{y}}_{ij}-{\bar{y}}_{j}\right)}^{2}}}$$

The statistic *k*_*ij*_ is a within-laboratory consistency evaluation comparing the standard deviation of the percent peak area value for a selected peak *j* within one laboratory *i* to the mean standard deviation for the different laboratories for peak *j*, *i*=1 to *p*, where *p* is the total number of laboratories and *s*_*ij*_ is the standard deviation from replicates for one sample within one laboratory.

The statistic *h*_*ij*_ is a between-laboratory consistency statistic that measures for a selected peak *j* the standardized version of the mean value of the percent peak area value obtained by laboratory i $\bar{y}$_ij_ from the grand mean for that peak $\bar{y}$_j_. This statistic provides a measure for any laboratory bias. The above obtained *k*_*i*_ and *h*_*i*_ values for the different peaks can be grouped per company and plotted with the critical values of *k* and *h* statistics at the significance level of 1% and 5%, explained in the ISO guideline 5725–2. Mandel's *k* and *h* values are only used as a graphical consistency technique and not to suggest outlier removal.

Test for homogeneity of variances: Cochran's test was implemented as a numerical technique to recognize outliers and/or stragglers in within-company variances. This test is calculated as:

(Eq. 3) $$C=\frac{s_{max}^{2}}{\sum_{i=1}^{P}s_{i}^{2}}$$

where $s_{max}^{2}$ and $s_{i}^{2}$ are the highest variance in the set and the variance from laboratory *i*, respectively. Outlier rejection criteria were taken from the ISO 5725–2 guideline, as well as critical C values. Variances significant at α = 0.01 (99% range) were considered as outliers and removed from the data set. Stragglers (values significant at α = 0.05, 95% range) on the other hand were kept in for further evaluations.

Test for outlying company means: Average of the results for the same level obtained by the companies were tested for outliers by the Grubb's tests where the single outlier test was applied meaning that it was investigated if there was one average, which was too high or too low, compared to the others:

(Eq. 4) $$G_{min}=\left(\bar{Y}-Y_{min}\right)/s$$

and

(Eq. 5) $$G_{max}=\left(Y_{max}-\bar{Y}\right)/s$$

with *Y*_*min*_ the smallest mean laboratory value, *Y*_*max*_ the largest mean laboratory value, $\bar{Y}\;\;$ the grand mean of all laboratories and *s* the standard deviation on all mean laboratory values. The absolute value of G is compared to the critical values for this test. A value larger than the critical value for the Grubb's test is considered an outlier.

## Supplemental Material

Szekrenyes et al Supplemental Data
