# Supplementary material for: Multi-Site N-glycan mapping study 1: Capillary electrophoresis – laser induced fluorescence
Source: MAbs. 2015 Oct 14;8(1):56–64. doi: 10.1080/19420862.2015.1107687 (PMC4966509; doi:10.1080/19420862.2015.1107687)
Supplement: Szekrenyes et al Supplemental Data [file kmab-08-01-1107687-s001.zip › Supplemental-information.pdf]

SUPLEMENTARY INFORMATION

DETAILED STUDY PROTOCOL

1. CAPILLARY ELECTROPHORESIS SYSTEM REQUIREMENTS

1.1. Instrumentation Systems and Software

Beckman Coulter PA 800 plus Pharmaceutical Analysis System, equipped with LIF detection, 32 Karat™ software v.9.1 (SCIEX Separations, Brea, CA).

1.2. Instrument Reagents and Consumables

Supplied to participants of the study by SCIEX Separations, Brea, CA.

Beckman Coulter PA 800 plus Carbohydrate Labeling and Analysis Kit (part number 477600), containing:

- N-CHO Capillary (2 each, 477601, lot M305010)
- N-Linked Carbohydrate Separation Gel Buffer (477623, lot M302376)
- LIF Performance Test Mixture (726022, lot M304365)

1.3. Additional Required Consumables

Supplied by participants.

Caps, Vial Buffer (Universal Rubber Vial Caps, Beckman part number A62250)

Vials (Universal Plastic Vial, Beckman part number A62251)

200 µl Micro Vials (Beckman part number 144709)

1.4. File Attachments Supplied to Participants

Sample Preparation and Analysis Protocols, PDF document

CE Method File: “CE Method 2psi 10 sec 060713.met”

Example data file: “Examples, Submission of Integrated Results, CE and UPLC.xlsx”

Expected Results (N-Glycan Partitioned Standards): “APTS high mannose.dat”; “APTS fucosyl biantennary.dat”; “APTS afucosyl biantennary.dat”

2. N-GLYCAN SAMPLE PREPARATION REQUIREMENTS

2.1. Reagents and Consumables for N-Glycan Sample Preparation

Supplied to participants of the study by ProZyme, Hayward, CA.

GS24-RX GlykoPrep Digestion Module 1 ea (lot 934 011-00ABD-A/B), containing:

- WS0253 Digestion (RX) Cartridges (24 Cartridges)
- WS0256-24 Immobilization Reagent Set
- WS0226-24 Denaturation Reagent (8 ml)
- WS0255-24 Blocking Reagent (1.5 ml)
- WS0259-24 Digestion Reagent Set
- WS0278-24 N-Glycanase (75 µl)
- WS0276 25x Digestion Buffer (0.7 ml)
- WS0229 Finishing Reagent (1 ml)
- Aluminum Sealing Film (1)

GS24-APTS GlykoPrep Rapid-Reductive-Amination APTS Labeling Module 1 ea (lot KG061313), containing:

- WS0299-24 APTS Solution (40 µl)
- WS0300-24 Reductant Solution (75 µl)
- WS0295-24 APTS Catalyst (75 µl)
- Aluminum Sealing Film (1)

GS24-C2 GlykoPrep APTS Cleanup Module 1 ea (lot KG061513), containing:

- WS0301-24 5x APTS Sample Load Buffer (15 ml)
- WS0263 Cleanup (CU) Cartridges (24 Cartridges)

Labware Pack (Cartridge Adapters & Associated Labware)

Consumables Pack (0.6-ml & 1.5-ml microcentrifuge vials)

2.2. Additional Required Reagents/Consumables/Equipment

Supplied by participants.

Centrifuge (capable of 50–1000 x *g*) and rotor for 1.5/2.0-ml microcentrifuge tubes

Heater and heat block that accommodates 0.2-ml PCR tubes

Centrifugal evaporator (*e.g.*, SpeedVac®) for drying released N-glycans prior to labeling

Vortexer

Fume hood (for labeling procedures)

pH meter

Top-loading balance

Analytical balance

Filtering apparatus, Millipore or equivalent with Type HA Filters

Pipettors & disposable tips (P5/P10, P200 and P1000)

Nitrile gloves

Ultrapure water

Acetonitrile

Volumetric pipettes, 5-ml

Glass graduated cylinder, 10-ml

Screw cap, glass storage vessel, 10-ml

2.3. Test Proteins and N-Glycan Standards

Supplied to participants of the study by ProZyme, Hayward, CA.

Protein Test Article solution (0.5 mg, 500 µl), lot ZS053013

GlykoPrep Training Protein (0.5 mg), Glyko hIgG GP Control, WS0162, lot W110230

APTS-(N-Glycan Test Article Solution), 50 µl, lot SM051813-2

CE System Glycan Standard Solution, 50 µl, lot SM052913

Glyko APTS-(Maltodextrin Ladder), 50 µl, lot BS060513

Glyko APTS-(Biantennary & High Mannose Partitioned Library), lot KG061413, containing:

APTS-(High Mannose N-Linked Glycan Library), 50 µl

APTS-(Fucosyl Biantennary N-Linked Glycan Library), 50 µl

APTS-(Afucosyl Biantennary N-Linked Glycan Library), 50 µl

3. N-GLYCAN SAMPLE PREPARATION PROCDURE

3.1. Digestion

3.1.1. Digestion Reagents and Supplies

GlykoPrep Training Protein Solution (1 mg/ml human IgG, prepared by dissolving 0.5 mg in 500 µl water). *GlykoPrep Training Protein Solution was previously used in the concurrent UPLC study for a 3-replicate training run, in which N-glycans were labeled with 2-AB and analyzed during UPLC system certification.*

Protein Test Article Solution (supplied at 1 mg/ml)

Digestion (RX) Cartridges (1 per sample, 4 total)

0.5-ml Microtube, screw cap, 0.2-ml PCR tubes, AssayMAP Cartridge Adaptors and flip-top, 1.75-ml Microcentrifuge tubes (supplied in the Labware Pack)

Denaturation Reagent

Finishing Reagent

Acetonitrile (100%, HPLC-grade), 50 µl/sample

Blocking Reagent (supplied with the 2-AB GlykoPrep Kit)

Digestion Buffer: prepare 400 µl of 1x Digestion Buffer by adding 16 µl of 25x Digestion Buffer to 384 µl of ultrapure water in a 0.6-ml microcentrifuge vial. Vortex to mix. *May be prepared up to one week before use, store at 2–8 ^{o}C.*

Enzyme Solution: Spin the N-Glycanase briefly to collect the contents in the base of the vial. Vortex the solution prior to use. Prepare 48 µl Enzyme Solution by adding 36 µl of Digestion Buffer to 12 µl of N-Glycanase in a 0.6-ml microcentrifuge vial. Invert gently multiple times to mix. *Prepare on the day of use, store at room temperature.*

3.1.2. Digestion Procedure

Dispense 75 µl of GlykoPrep Training Protein Solution to a 0.6-ml vial (the Control).

Dispense 200 µl of Protein Test Article Solution to a 1.5-ml vial (the Sample).

Denature

The Denaturation Reagent (6 M Guanidine) is viscous, mix well prior to use.

1.a Add 75 µl of Denaturation Reagent to the Control. Pipet up and down to mix well. This will be enough for a single Cartridge.

1.b Add 200 µl of Denaturation Reagent to the Sample. Pipet up and down to mix well. This will be enough for three replicates with some overfill.

1.c Allow to incubate at room temperature for at least 5 minutes.

Prepare

2.a Number each RX Cartridge (1 Control and 3 Sample Replicates). Prepare a Basic Assembly for each by nesting the numbered RX Cartridges into 0.5-ml screw cap Microtubes.

2.b Pipet 50 µl of 100% Acetonitrile into the Sample Cup of each RX Cartridge in the Basic Assemblies.

2.c Place the Basic Assemblies in the centrifuge and spin at 300 x *g* for 3 minutes.

Proceed through the Prepare, Equilibrate and Load steps without interruption, as evaporation can lead to airlock.

Equilibrate

3.a Pipet 150 µl of Denaturation Reagent into the Sample Cup of each RX Cartridge.

3.b Spin at 1000 x *g* for 2 minutes.

Load

4.a Load 100 µl of the denatured Control and Sample Replicates into the Sample Cup of each RX Cartridge.

4.b Empty the flow-through by lifting each RX Cartridge and pouring out the liquid collected in the Microtube below. Dispose of the liquid as organic waste and return each RX Cartridge to its Microtube.

4.c Spin at 50 x *g* until all Sample Cups are empty (~15 minutes).

Check that Sample Cups are empty before proceeding or yield will be reduced.

Block

5.a Pipet 50 µl of Blocking Reagent into the Sample Cup of each RX Cartridge.

5.b Empty the flow-through (as described in 4.b).

5.c Spin at 300 x *g* for 3 minutes.

Wash

6.a Pipet 50 µl of Digestion Buffer into the Sample Cup of each RX Cartridge.

6.b Empty the flow-through (as described in 4.b).

6.c Spin at 300 x *g* for 3 minutes.

Load N-Glycanase

7.a Number four 0.2-ml PCR tubes for the Control and Sample Replicates.

7.b Prepare four "Tips Wet" Adapter Assemblies by nesting the numbered PCR tubes into AssayMAP Cartridge Adaptors and 1.75-ml Microcentrifuge tubes.

7.c Pipet 10 µl of Enzyme Solution into the Sample Cup of each RX Cartridge in the "Tips Wet" Adaptor Assemblies.

7.d Transfer the RX Cartridges from step 6.b into the corresponding "Tips Wet" Adaptor Assemblies. Dispose of Microtubes and flow-through from 6.b.

7.e Spin at 300 x *g* for 3 minutes; **do not discard flow-through**.

Incubate

8. Transfer the RX Cartridges/PCR tube portions (Incubation Assembly) to the equilibrated 45 ^{o}C PCR heat block and incubate for 30 minutes.

Elute (and Finish)

9.a Remove the Incubation Assemblies from the heat block and reinsert into the Adapters/1.75-ml Microcentrifuge tubes to form "Tips Wet" Adaptor Assemblies again.

9.b Pipet 15 µl of Finishing Reagent into the Sample Cup of each RX Cartridge.

9.c Spin at 300 x *g* for 3 minutes.

9.d Remove RX Cartridges from the PCR tubes. The eluted N-glycans are in the PCR tubes; DO NOT DISCARD.

10. Close the cap on each PCR tube and incubate on the equilibrated 45 ^{0}C heat block for 10 minutes.

11. Open the PCR tubes, return them to the "Tips Wet" Adaptor Assemblies (now minus the RX Cartridges). Dry in a centrifugal evaporator (SpeedVac, heat setting turned to the off position) until fully dry (~30 minutes).

Used RX Cartridges may be discarded. After removing the PCR tubes from the Heat Block, adjust the temperature setting to 50 ℃. The N-glycans are condensed into a pellet small enough to be dissolved by 4.5 µl of APTS Labeling Reagent in the next step.

3.2. APTS Labeling of Released N-Glycans

3.2.1. Preparation of APTS Labeling Reagent

Just prior to use, allow the APTS Solution, APTS Catalyst and Reductant Solution (supplied with GS24-APTS) to come to room temperature in the sealed desiccant bag. Then invert the vials to mix.

In a separate vial, prepare a master mixture of APTS Labeling Reagent by adding 5 µl of APTS Solution, 12.5 µl of APTS Catalyst, and 5 µl of Reductant Solution. Cap tightly and vortex on high for 10 seconds to mix.

APTS Labeling Reagent must be prepared just prior to use. Catalyst and Reductant Solutions are hygroscopic; minimize exposure to air and protect from exposure to light. Return any unused APTS Labeling Solution and Reductant Solution to the desiccant-containing bag and store at -20 ℃. The APTS Labeling Reagent (APTS Solution + APTS Catalyst + Reductant) will generate bubbles over time, so spin down in a centrifuge and use immediately. If the solution begins to bubble during use, spin down again.

3.2.2. APTS Labeling Procedure

1.a Add 4.5 µl of APTS Labeling Reagent to the Control and each Sample Replicate (dried N-glycans in 0.2-ml PCR tubes).

1.b Return the PCR tubes to the “Tips Wet” Adapter Assemblies (minus the RX Cartridges) and spin at 300 x *g* for 1 minute to ensure the liquid is collected at the bottom of the wells.

Before use, be sure each heat block has equilibrated to 50 ℃; a thermometer may be placed in the corner thermometer well of the heat block to test the temperature.

Incubate

2.a Close the cap on each PCR tube and transfer it to the heat block.

2.b Incubate at 50 ºC on the equilibrated heat block for 1 hour.

2.c Remove the PCR tubes from the heat block and allow to cool to room temperature (~5 minutes).
2.d In a fume hood, open each PCR tube.

This is a good time to prepare the APTS Sample Load Buffer; directions may be found in the next section, Cleanup. It is normal for condensate to collect on the underside of the lid. DO NOT centrifuge the tube to collect the condensate. ***Proceed immediately to Cleanup.***

3.3. Cleanup of APTS-Labeled N-Glycans

3.3.1. APTS Cleanup Reagents & Supplies

N-Glycan Control and Sample Replicates from APTS Labeling (in PCR tubes)
CU Cartridges (supplied with GS24-C2, 1 per N-glycan sample)
AssayMAP Cartridge Adaptors and flip-top, 1.75-ml Microcentrifuge tubes, 2 per sample (supplied with the Labware Pack)
Acetonitrile (100%)
Ultrapure water
Volumetric pipettes, 5-ml
Glass graduated cylinder, 10-ml
Screw cap, glass storage vessel, 10-ml
5x APTS Sample Load Buffer (supplied with the APTS GlykoPrep Kit)
Prepare 5 ml of 1x APTS Sample Load Buffer by adding 1 ml of 5x APTS Sample Load Buffer to a small, glass graduated cylinder. Bring the volume up to 5 ml with 100% Acetonitrile. Transfer to a glass storage vessel, cap tightly and swirl thoroughly to ensure complete dissolution. It is important to prevent evaporation of the Acetonitrile which would affect performance of the HILIC separation during cleanup.

3.3.2. APTS Cleanup Procedure

Prepare one "Tips Free" Assembly per Sample Replicate by nesting a CU Cartridge into an AssayMAP Cartridge Adaptor and a 1.75-ml Microcentrifuge tube.

Load

1.a Add 200 µl of APTS Sample Load Buffer to the Control and each Sample Replicate in the PCR tubes. Pipet up and down to mix.

1.b Transfer the Control and each Sample Replicate into the Sample Cup of a CU Cartridge in a "Tips Free" Assembly.

1.c Spin at 300 x *g* for 3 minutes or until the Sample Cup of each CU Cartridge is empty.

Transfer the mixture as quickly as possible because Acetonitrile solution has very low viscosity and may drip from the pipette tip; each sample may be pipetted in multiple rounds in order to achieve a quantitative transfer.

Wash

2.a Pipet 200 µl of APTS Sample Load Buffer into the Sample Cup of each CU Cartridge in the "Tips Free" Assembly.

2.b Spin at 300 x *g* for 3 minutes.

Elute

3.a Number a clean 1.75-ml Microcentrifuge tube for the Control and each Sample Replicate.

3.b Transfer each CU Cartridge with its Adaptor to the new, numbered Microcentrifuge tube.

3.c Pipet 100 µl of water into the Sample Cup of each CU Cartridge.

3.d Spin at 100 x *g* for 10 minutes.

3.e Then spin at 300 x *g* for 3 minutes to elute all liquid from the CU Cartridge.

The 1.75-ml Microcentrifuge tubes now contain the APTS-labeled N-Glycans with free dye and buffer salts removed; **DO NOT DISCARD**.

Check each CU Cartridge to make sure the Sample Cup is empty. Otherwise continue to spin at 300 x g for 3-minute intervals. Used CU Cartridges may be discarded. Adapters are reusable, DO NOT DISCARD. N-Glycan Samples are now ready to be analyzed. If not analyzed immediately, store sealed at -20 ºC in the dark.

4. PROCDURE FOR SEPARATION OF APTS-LABELED N-GLYCANS BY CAPILLARY ELECTROPHORESIS

4.1. Materials & Reagents

N-CHO Coated Capillary (total length 60 cm, effective length 50 cm)

Carbohydrate Separation Buffer

Beckman Micro Vials

Beckman Universal Plastic Vials and Rubber Caps

LIF Performance Test Mixture

CE System Glycan Standard (CE System Suitability Standard) Solution (50 µl)

Includes a mixture of APTS-labeled N-glycans, some are which are known to be difficult to resolve using CE (G0FB, G1, G1F, G2).

APTS-(Maltodextrin Ladder) Solution, 50 µl

APTS-labeled N-Glycan Samples (prepared previously from the Protein Test Article and the GlykoPrep Training Protein)

APTS-(N-Glycan Test Article) Solution, 50 µl

Glyko APTS-(Biantennary & High Mannose Partitioned N-Linked Glycan Library) Solution (50 µl)

Includes the APTS-(High Mannose N-Linked Glycan Library), the APTS-(Fucosyl Biantennary N-Linked Glycan Library) and the APTS-(Afucosyl Biantennary N-Linked Glycan Library)

Electropherograms of the APTS-(Biantennary & High Mannose Partitioned N-Linked Glycan Library), with migration time and peak identifications. These were supplied to participants (see section 1.3) for the purpose of N-Glycan Standard peak identification.

4.2. Preparing Samples for CE Analysis

Pipette each APTS-labeled N-Glycan Sample up and down several times to mix thoroughly, and then transfer the entire sample from each 1.75-ml Microcentrifuge tube to a Beckman Micro Vial. Insert each Micro Vial into a Universal Plastic Vial and cap with a Universal Rubber Cap. Position on the Sample Tray. Pipette the APTS-labeled N-Glycan Test Article Solution up and down several times to mix thoroughly, and then transfer the entire sample to a Beckman Micro Vial. Insert the Micro Vial into a Universal Plastic Vial and cap with a Universal Rubber Cap. Position on the Sample Tray.

If samples are frozen, allow to equilibrate to room temperature before use.

4.3. Preparing Standards for CE Analysis

Pipette each standard up and down several times to mix thoroughly and then transfer the entire volume to a Beckman Micro Vial. Insert each Micro Vial into a Universal Plastic Vial and cap with a Universal Rubber Cap. Position on the Sample Tray. For the water blank, pipet 100 µl of ultrapure water into a Beckman Micro Vial. Insert each Micro Vial into a Universal Plastic Vial and cap with a Universal Rubber Cap. Position on the Sample Tray.

4.4. Injection Sequence for CE System Certification

1. Water blank
2. APTS-(Maltodextrin Ladder) Solution
3. APTS-(High Mannose N-Linked Glycan Library)
4. APTS-(Fucosyl Biantennary N-Linked Glycan Library)
5. APTS-(Afucosyl Biantennary N-Linked Glycan Library)
6. CE System Glycan Standard Solution (rep 1)
7. CE System Glycan Standard Solution (rep 2)
8. CE System Glycan Standard Solution (rep 3)
9. APTS-(Maltodextrin Ladder) Solution

4.5. Injection Sequence for Analysis of Test Article Preparations

1. Water blank
2. APTS-(Maltodextrin Ladder) Solution
3. Control Glycoprotein N-Glycans (GlykoPrep control)
4. APTS-(High Mannose N-Linked Glycan Library) Solution
5. APTS-(Fucosyl Biantennary N-Linked Glycan Library) Solution
6. APTS-(Afucosyl Biantennary N-Linked Glycan Library) Solution
7. APTS-(Maltodextrin Ladder) Solution
8. Protein Test Article (replicate 1, analysis 1)
9. Protein Test Article (replicate 1, analysis 2)
10. Protein Test Article (replicate 1, analysis 3)

11. APTS-(Maltodextrin Ladder) Solution
12. Protein Test Article (replicate 2, analysis 1)
13. Protein Test Article (replicate 2, analysis 2)
14. Protein Test Article (replicate 2, analysis 3)
15. APTS-(Maltodextrin Ladder) Solution
16. Protein Test Article (replicate 3, analysis 1)
17. Protein Test Article (replicate 3, analysis 2)
18. Protein Test Article (replicate 3, analysis 3)
19. APTS-(Maltodextrin Ladder) Solution
20. APTS-(Glycan Test Article) Solution (analysis 1)
21. APTS-(Glycan Test Article) Solution (analysis 2)
22. APTS-(Glycan Test Article) Solution (analysis 3)
23. APTS-(Maltodextrin Ladder) Solution
