# Supplementary material for: Multi-Site N-glycan mapping study 1: Capillary electrophoresis – laser induced fluorescence
Source: MAbs. 2015 Oct 14;8(1):56–64. doi: 10.1080/19420862.2015.1107687 (PMC4966509; doi:10.1080/19420862.2015.1107687)
Supplement: Szekrenyes et al Supplemental Data [file kmab-08-01-1107687-s001.zip › Supplemental-data.pdf]

SUPPLEMENTAL DATA

Figure S1. Results for site A

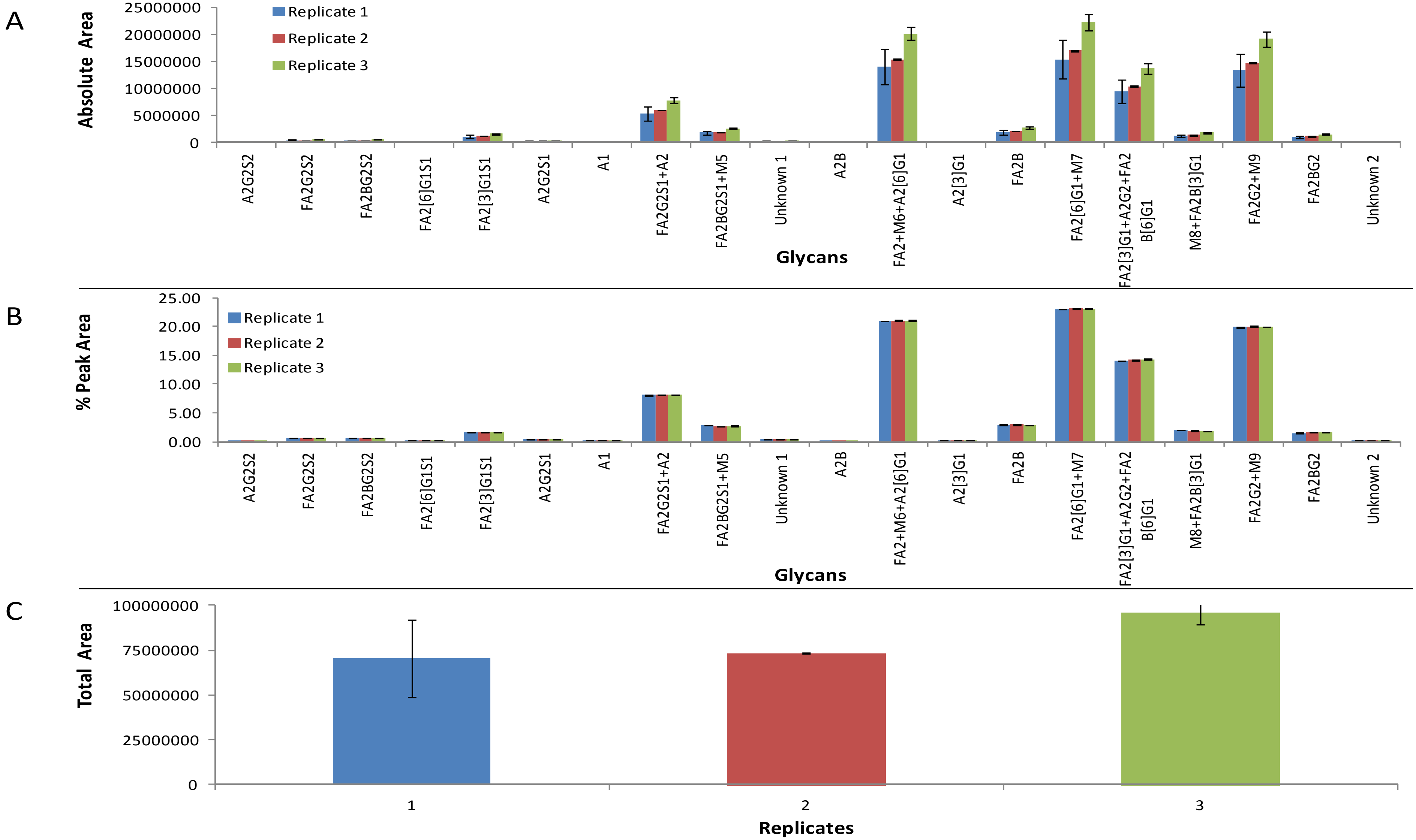

Figure S1. Statistical analysis of site A. Three chemical replicates were prepared; three injections were performed for each replicate. (A) Absolute peak area for each integrated peak. (B) Relative peak areas for each integrated peak. (C) Total peak area of each replicate.

Figure S2. Results for site B

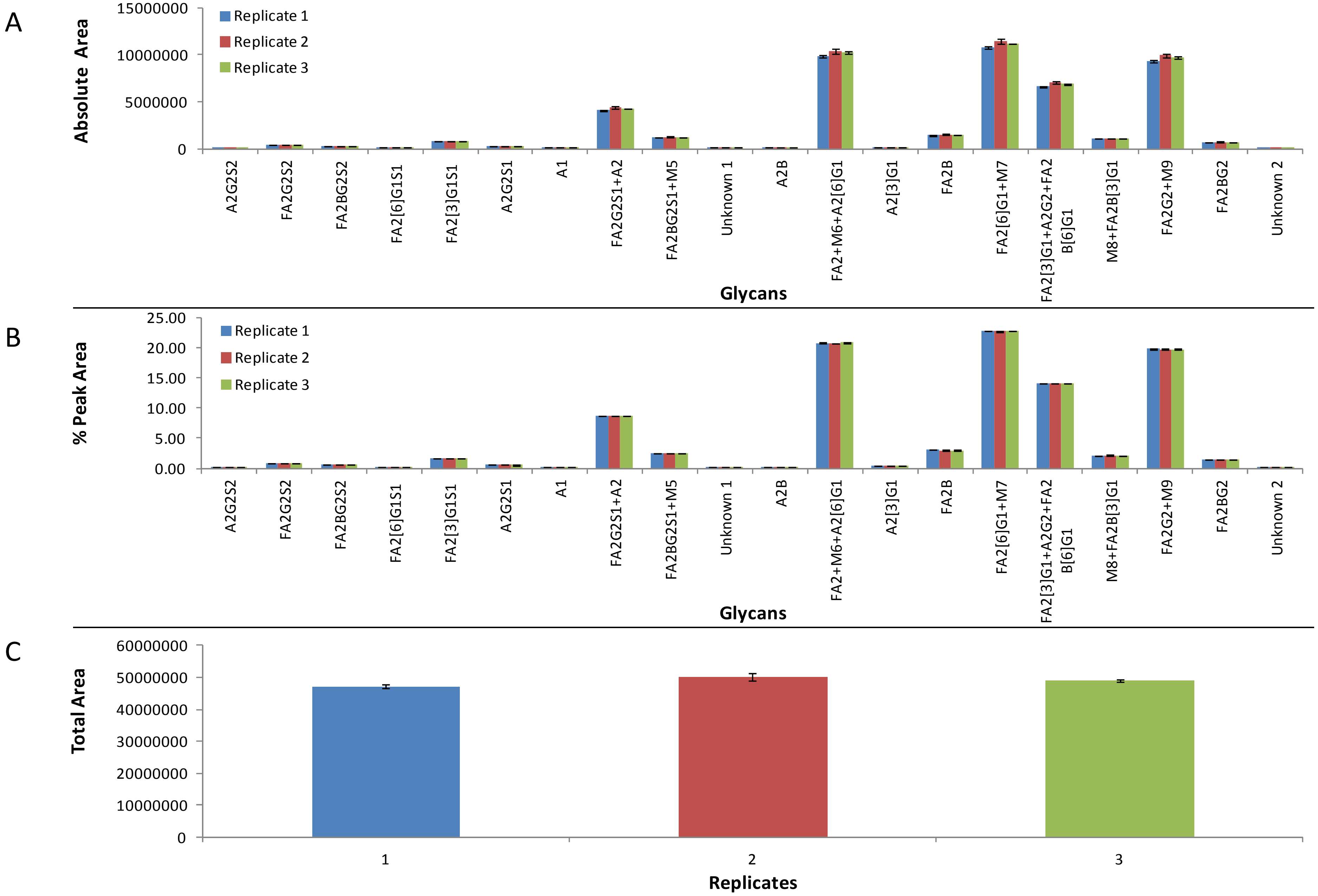

Figure S2. Statistical analysis of site B. Three chemical replicates were prepared; three injections were performed for each replicate. (A) Absolute peak area for each integrated peak. (B) Relative peak areas for each integrated peak. (C) Total peak area of each replicate.

Figure S3. Results for site D

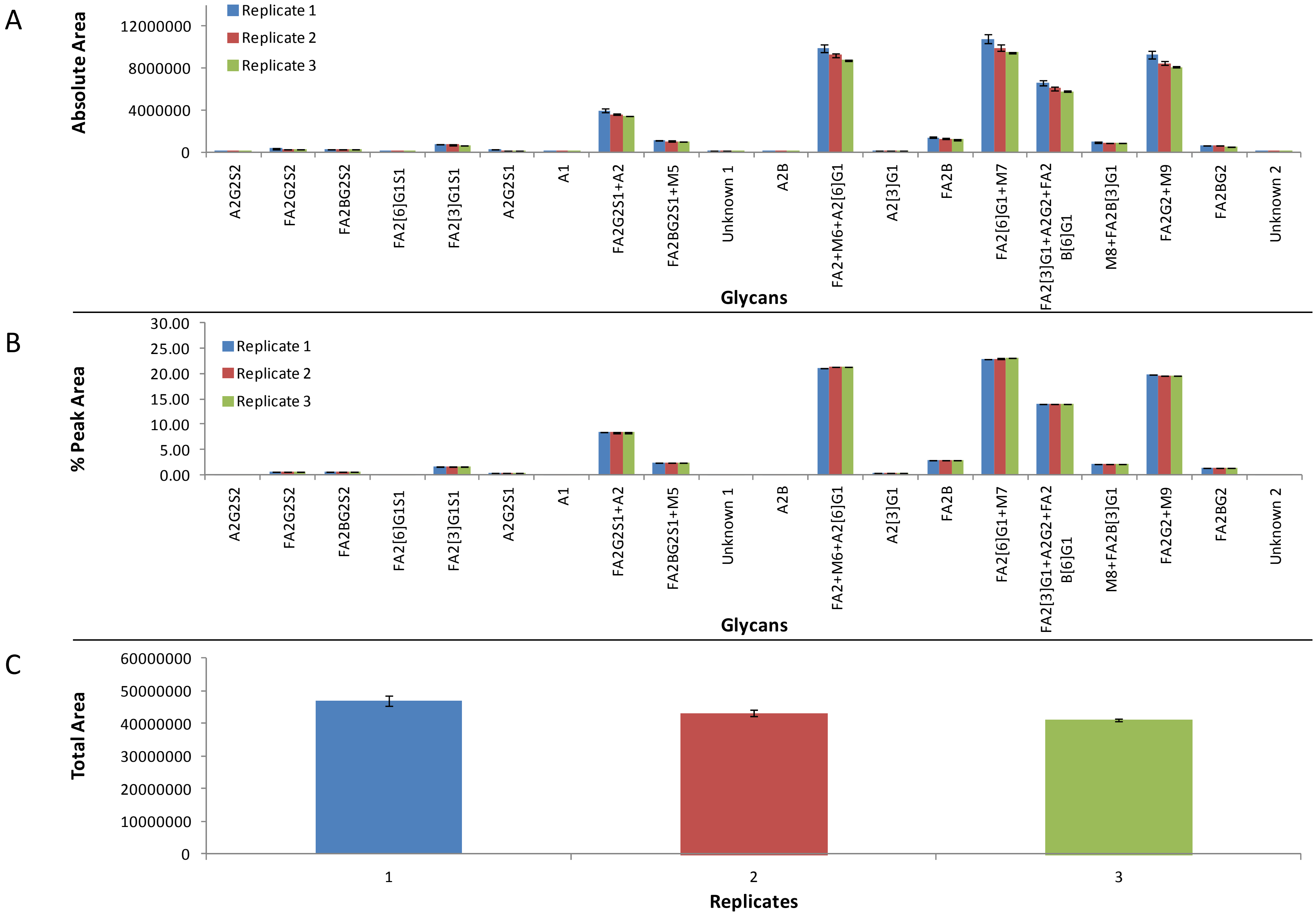

Figure S3. Statistical analysis of site D. Three chemical replicates were prepared; three injections were performed for each replicate. (A) Absolute peak area for each integrated peak. (B) Relative peak areas for each integrated peak. (C) Total peak area of each replicate.

Figure S4. Results for site E

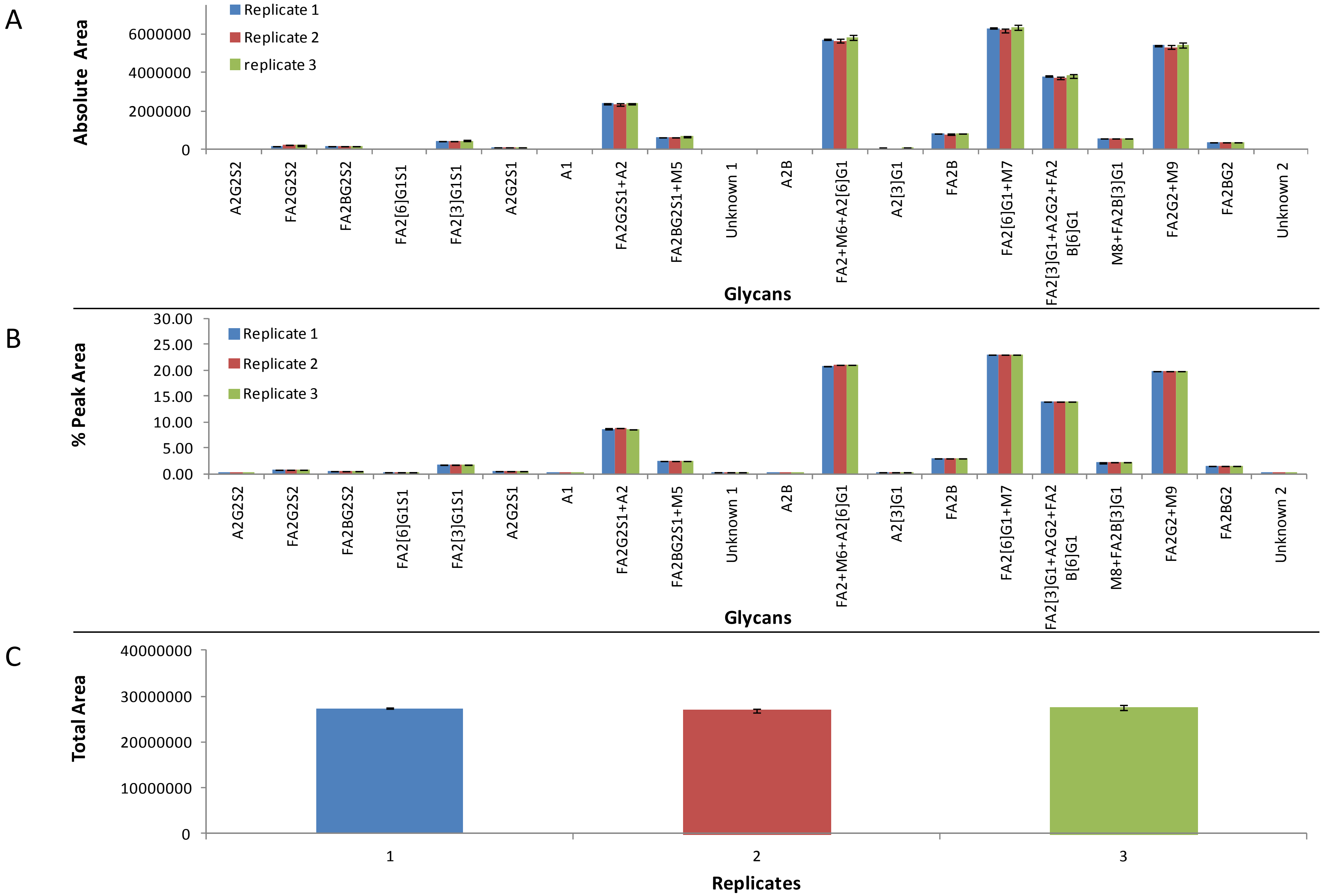

Figure S4. Statistical analysis of site E. Three chemical replicates were prepared; three injections were performed for each replicate. (A) Absolute peak area for each integrated peak. (B) Relative peak areas for each integrated peak. (C) Total peak area of each replicate.

Figure S5. Results for site F

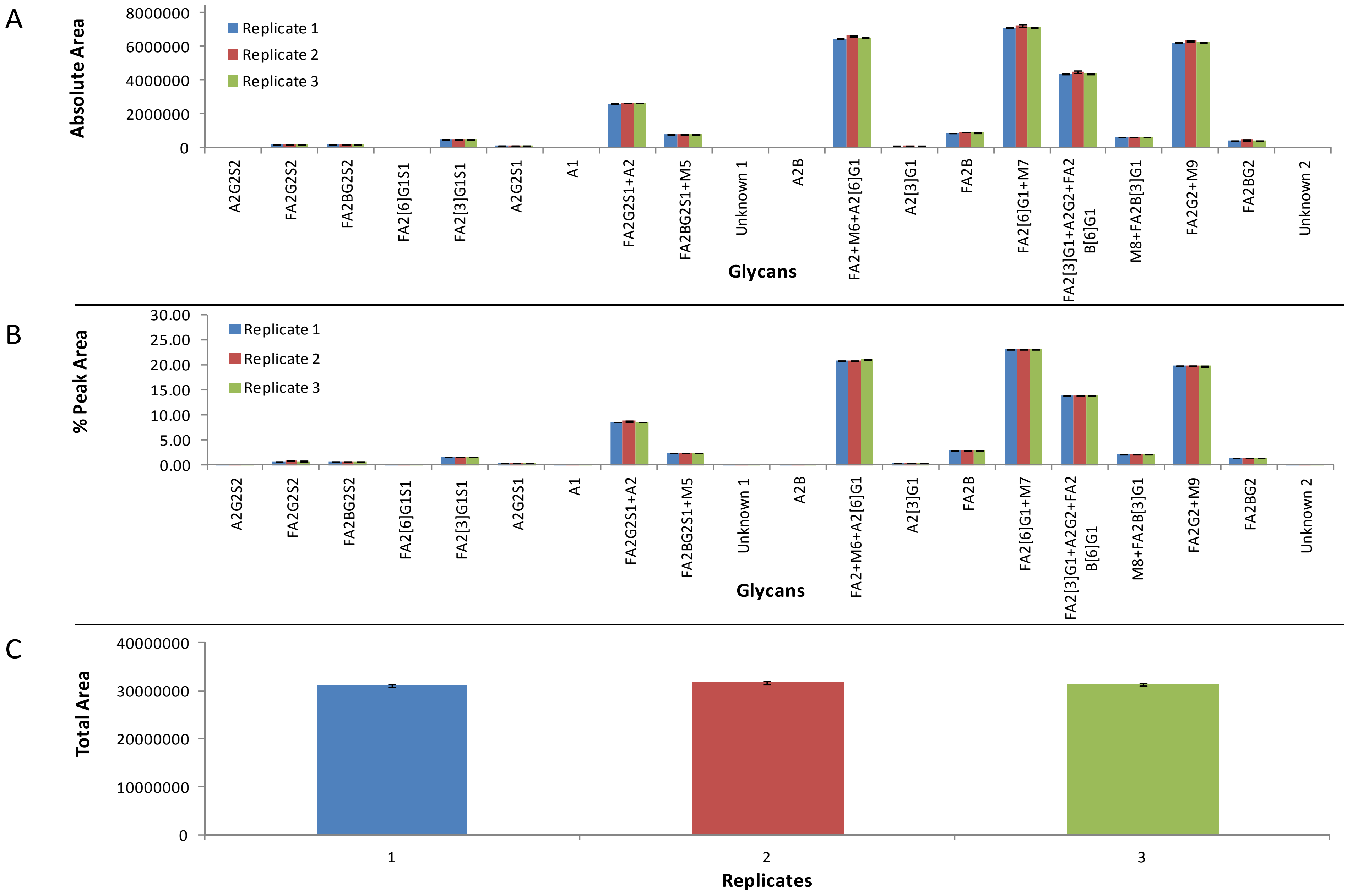

Figure S5. Statistical analysis of site F. Three chemical replicates were prepared; three injections were performed for each replicate. (A) Absolute peak area for each integrated peak. (B) Relative peak areas for each integrated peak. (C) Total peak area of each replicate.

Figure S6. Results for site G

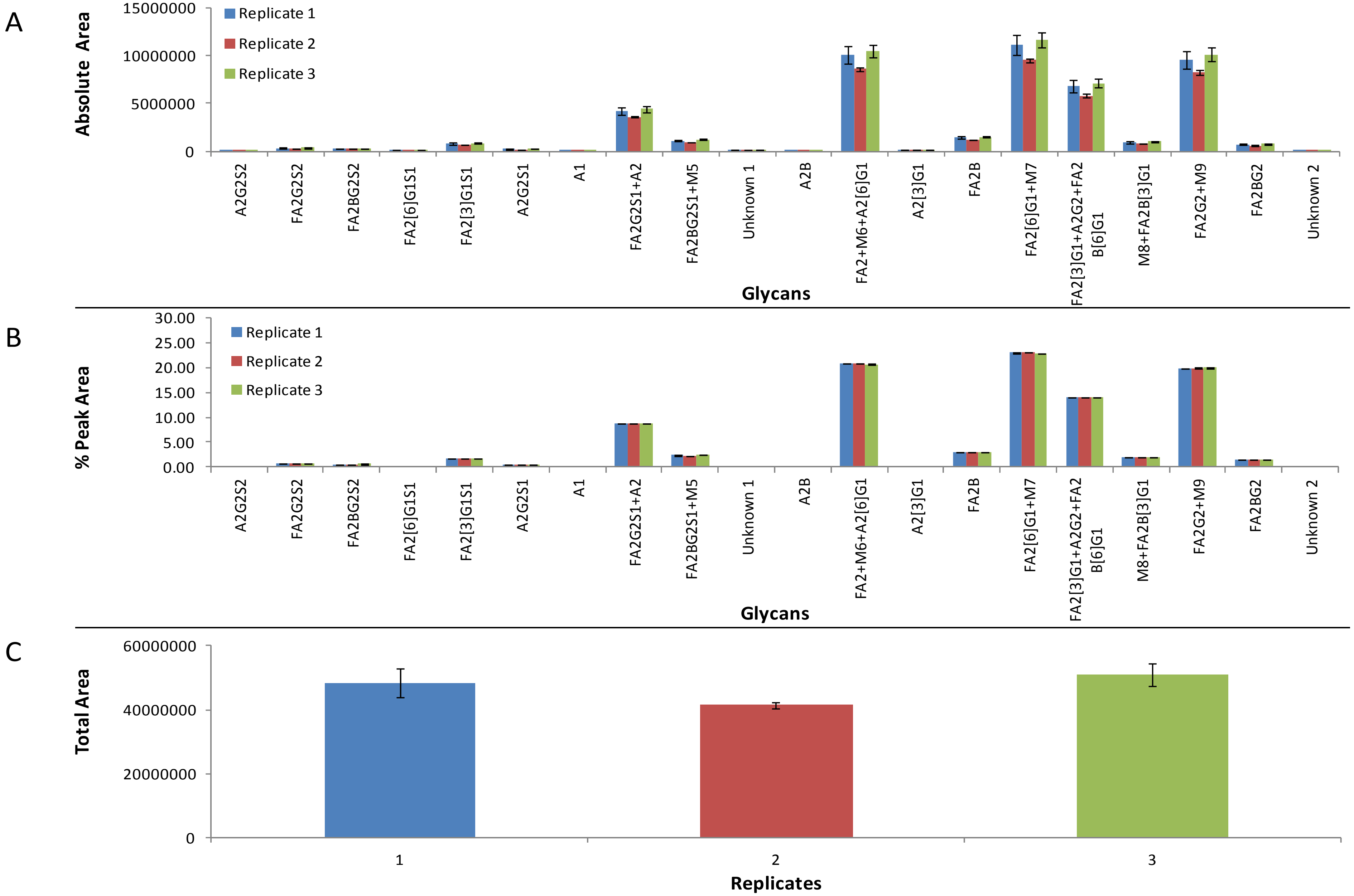

Figure S6. Statistical analysis of site G. Three chemical replicates were prepared; three injections were performed for each replicate. (A) Absolute peak area for each integrated peak. (B) Relative peak areas for each integrated peak. (C) Total peak area of each replicate.

Figure S7. Results for site H

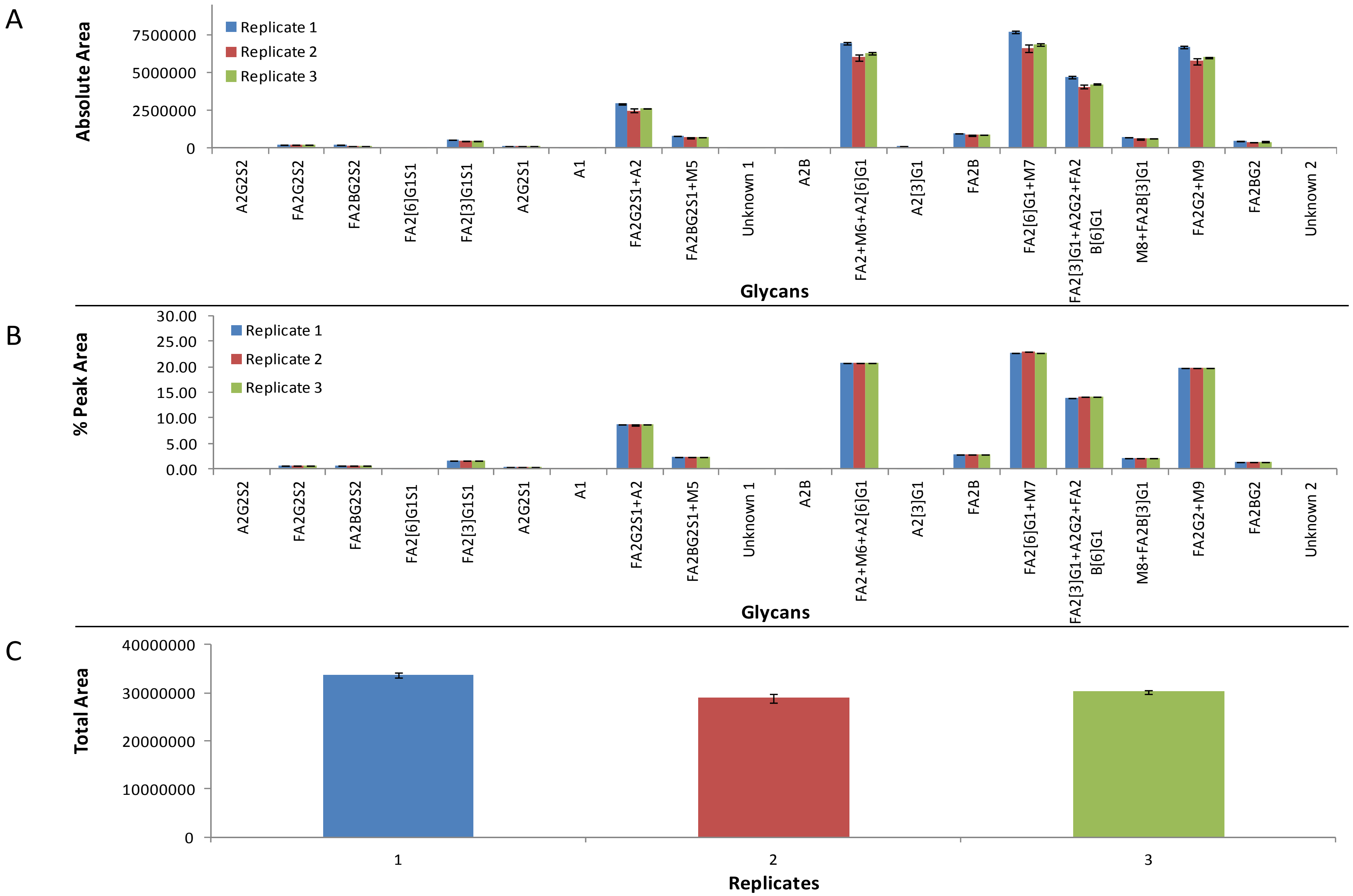

Figure S7. Statistical analysis of site H. Three chemical replicates were prepared; three injections were performed for each replicate. (A) Absolute peak area for each integrated peak. (B) Relative peak areas for each integrated peak. (C) Total peak area of each replicate.

Figure S8. Results for site I

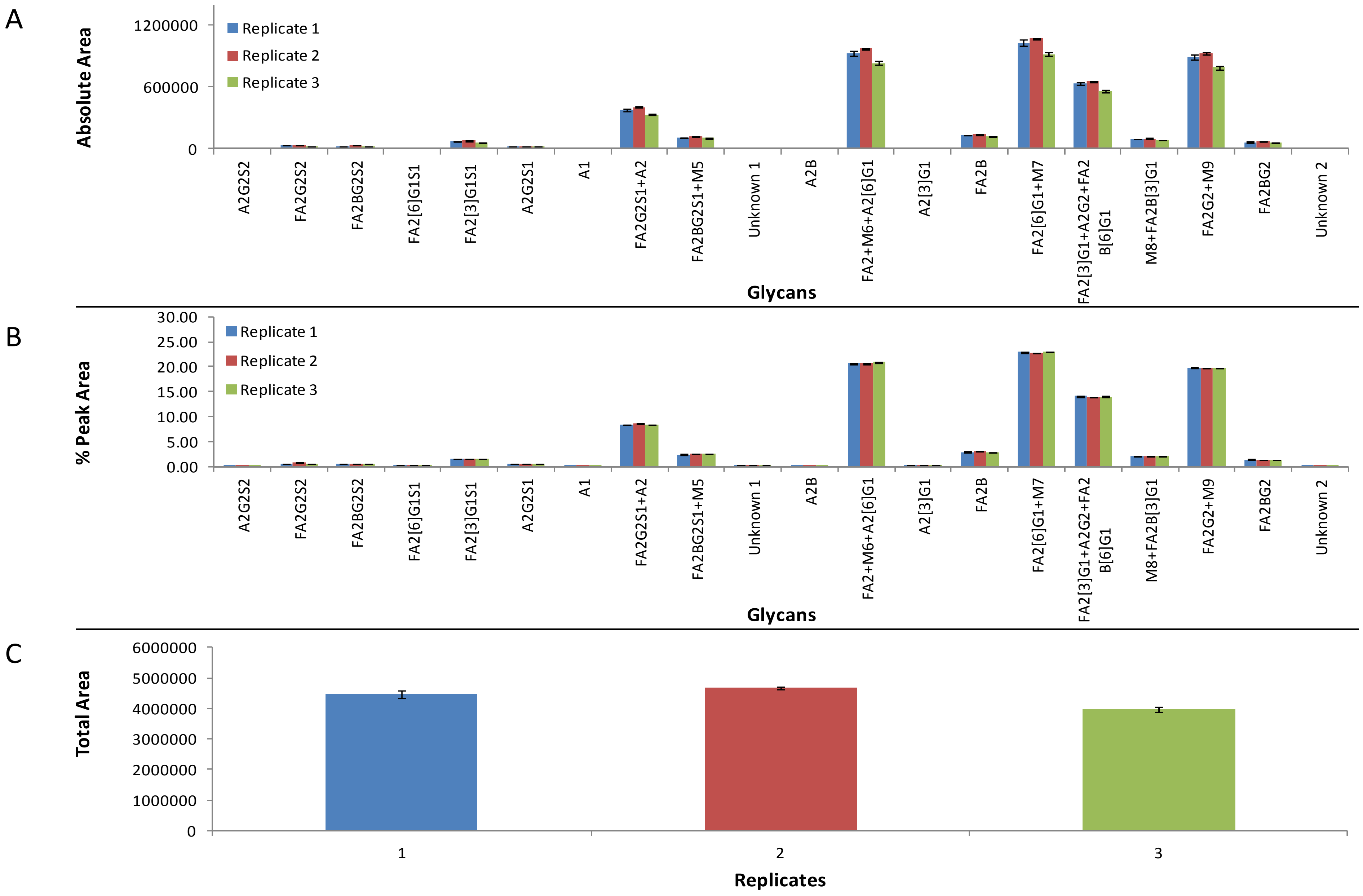

Figure S8. Statistical analysis of site I. Three chemical replicates were prepared; three injections were performed for each replicate. (A) Absolute peak area for each integrated peak. (B) Relative peak areas for each integrated peak. (C) Total peak area of each replicate.

Figure S9. Results for site J

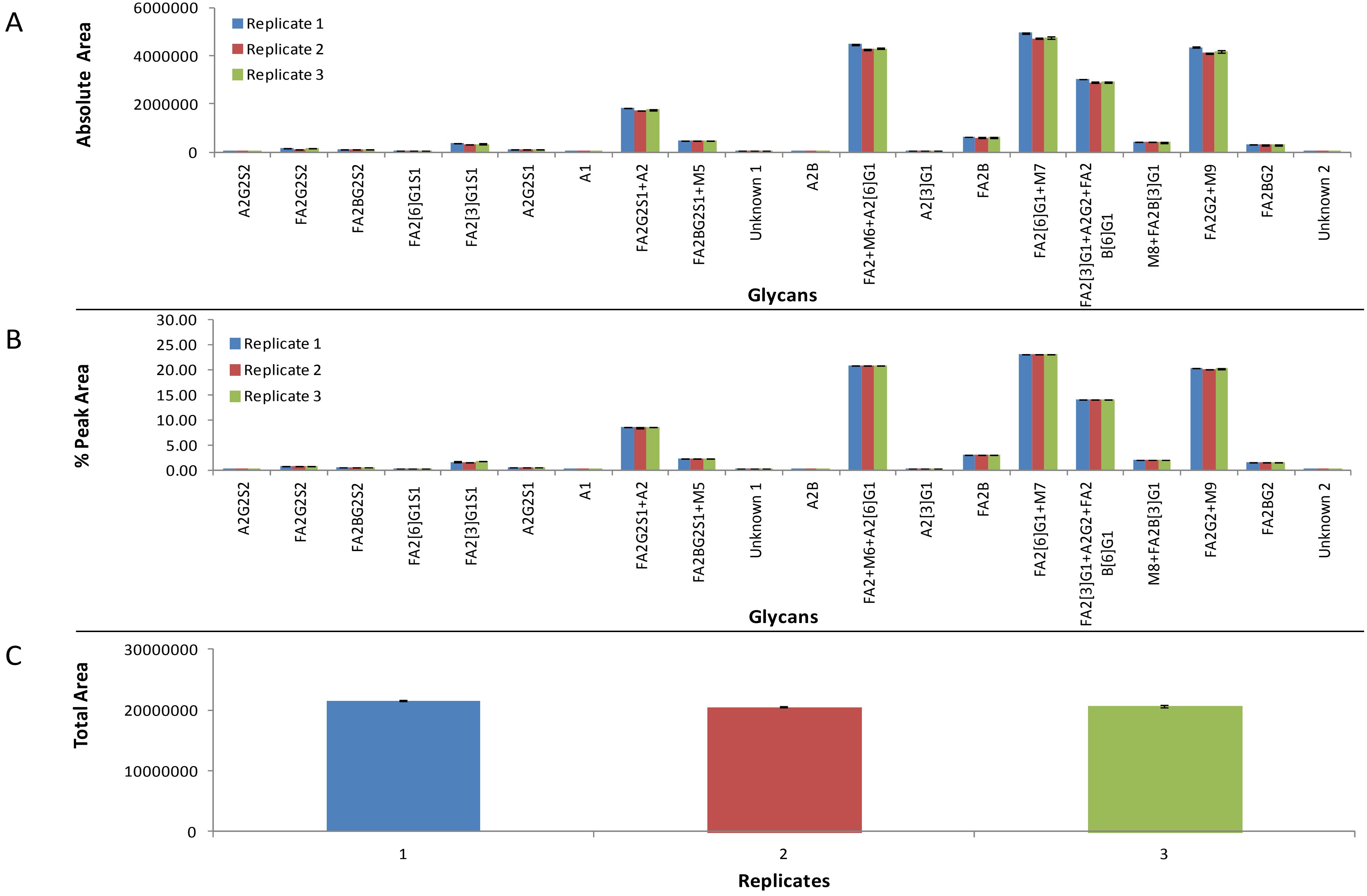

Figure S9. Statistical analysis of site J. Three chemical replicates were prepared; three injections were performed for each replicate. (A) Absolute peak area for each integrated peak. (B) Relative peak areas for each integrated peak. (C) Total peak area of each replicate.

Figure S10. Results for site K

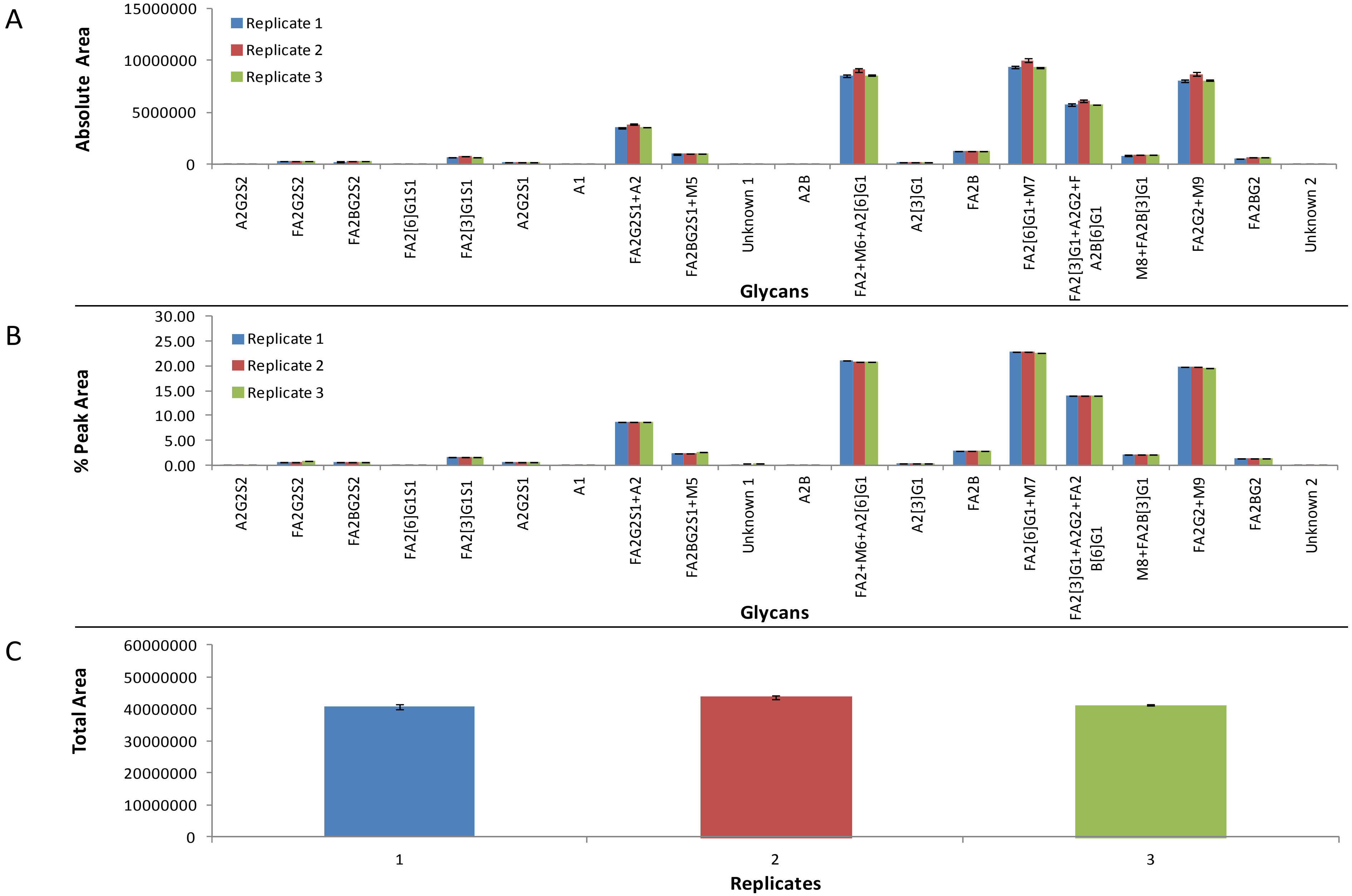

Figure S10. Statistical analysis of site K. Three chemical replicates were prepared; three injections were performed for each replicate. (A) Absolute peak area for each integrated peak. (B) Relative peak areas for each integrated peak. (C) Total peak area of each replicate.

Figure S11. Results for site L

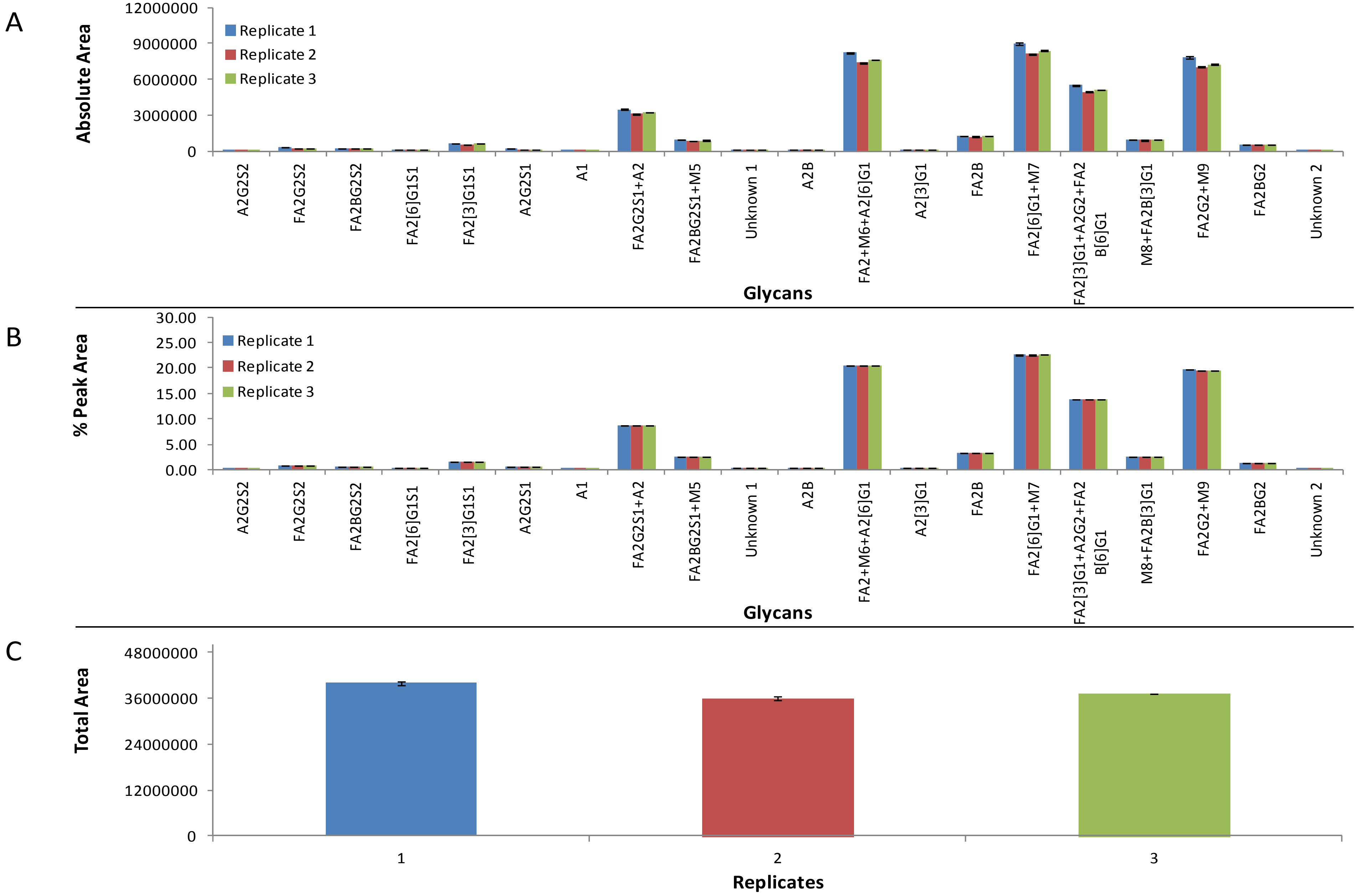

Figure S11. Statistical analysis of site L. Three chemical replicates were prepared; three injections were performed for each replicate. (A) Absolute peak area for each integrated peak. (B) Relative peak areas for each integrated peak. (C) Total peak area of each replicate.

Figure S12. Results for site M

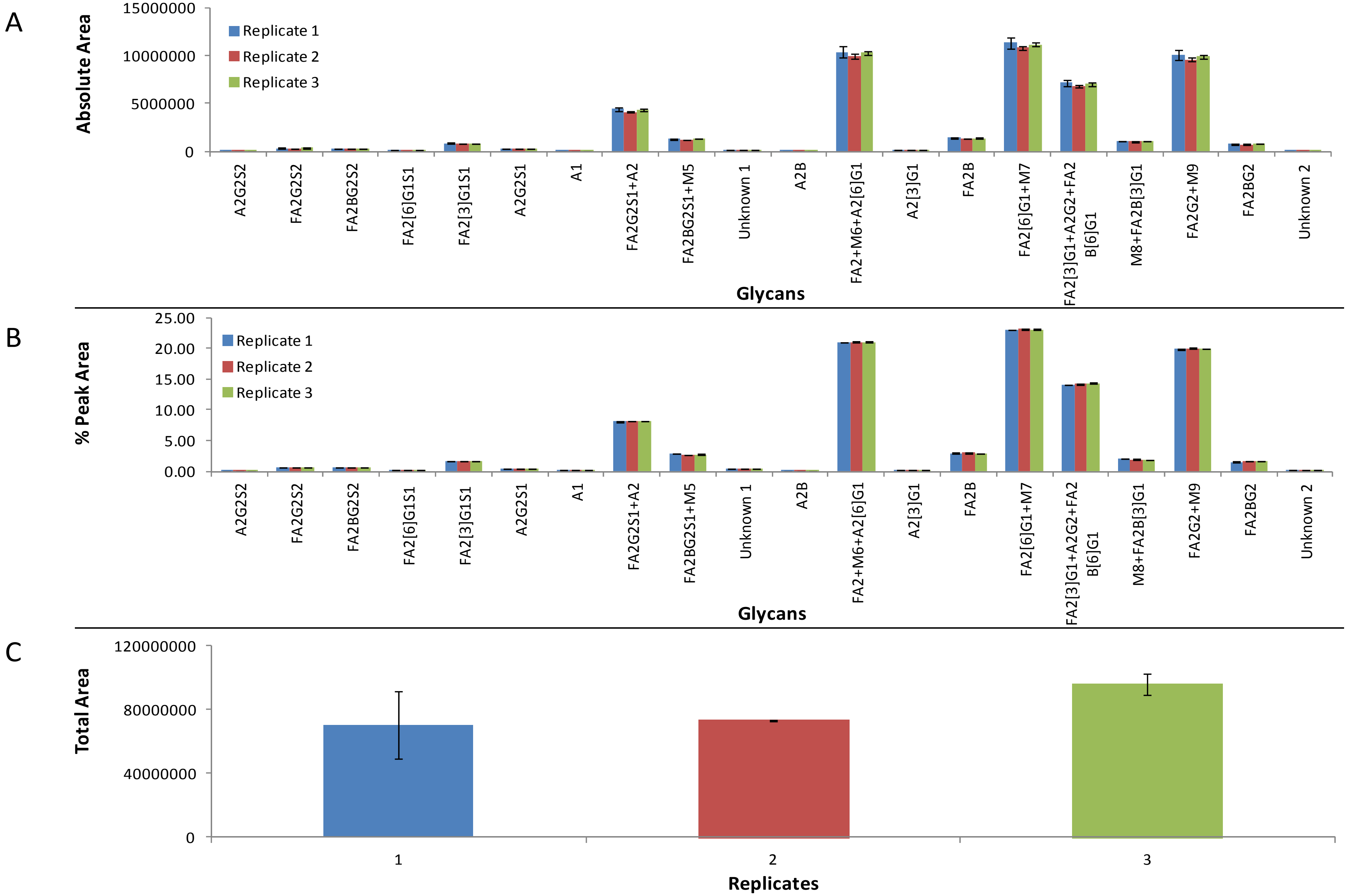

Figure S12. Statistical analysis of site M. Three chemical replicates were prepared; three injections were performed for each replicate. (A) Absolute peak area for each integrated peak. (B) Relative peak areas for each integrated peak. (C) Total peak area of each replicate.

Figure S13. Results for site N

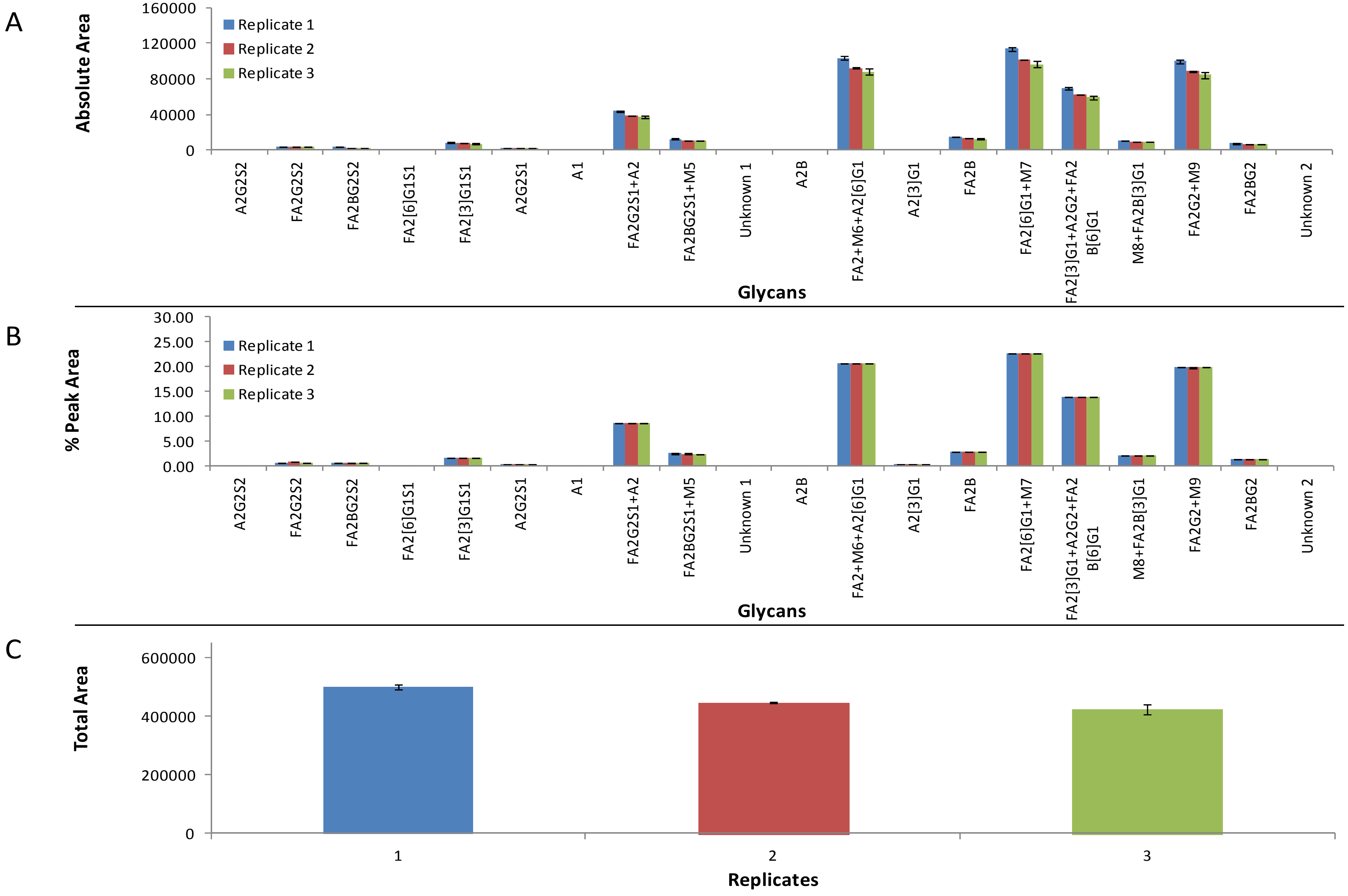

Figure S13. Statistical analysis of site N. Three chemical replicates were prepared; three injections were performed for each replicate. (A) Absolute peak area for each integrated peak. (B) Relative peak areas for each integrated peak. (C) Total peak area of each replicate.

Figure S14. Results for site O

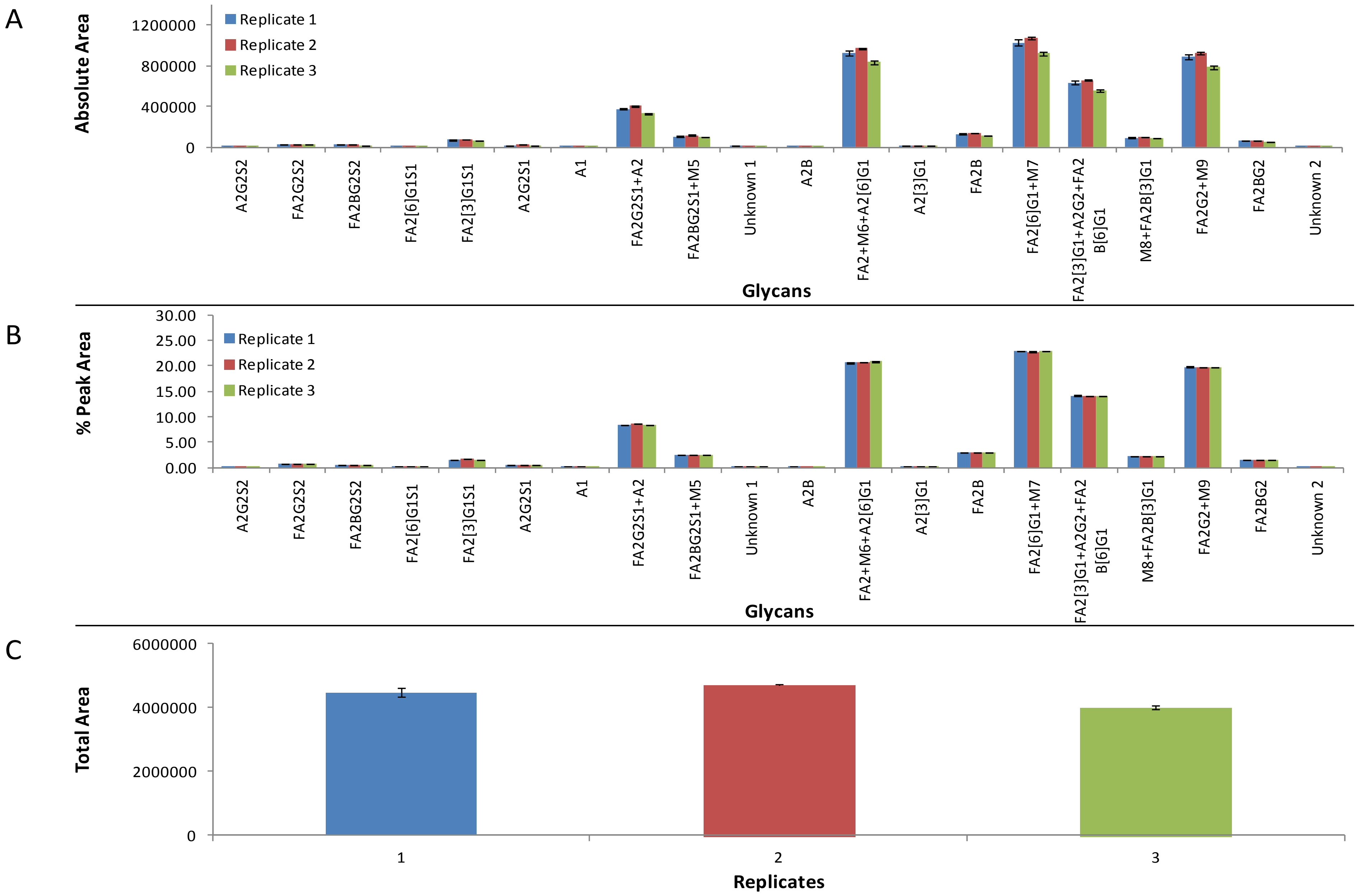

Figure S14. Statistical analysis of site O. Three chemical replicates were prepared; three injections were performed for each replicate. (A) Absolute peak area for each integrated peak. (B) Relative peak areas for each integrated peak. (C) Total peak area of each replicate.

Figure S15. Results for site P

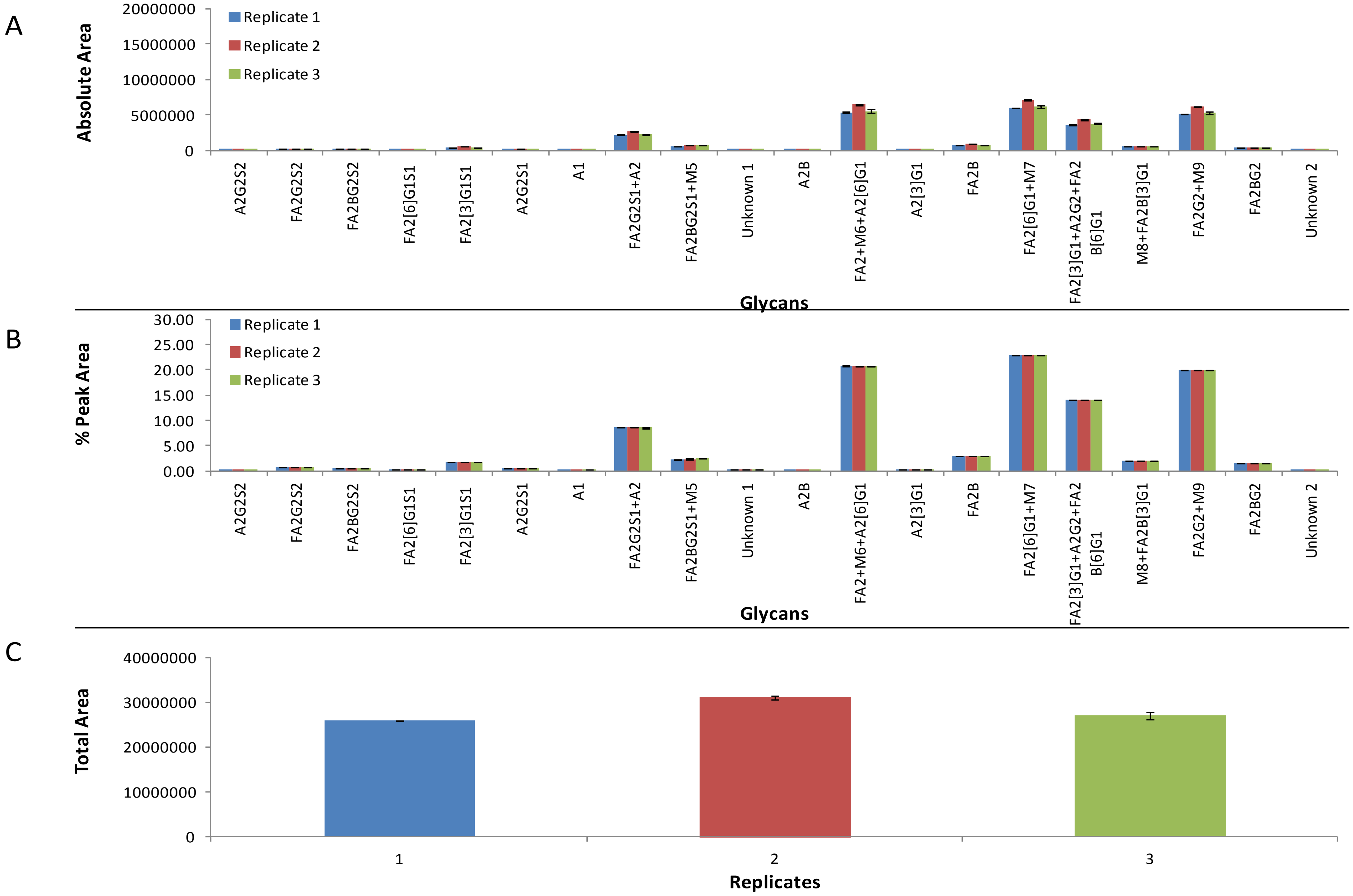

Figure S15. Statistical analysis of site P. Three chemical replicates were prepared; three injections were performed for each replicate. (A) Absolute peak area for each integrated peak. (B) Relative peak areas for each integrated peak. (C) Total peak area of each replicate.

Figure S16. Results for site S

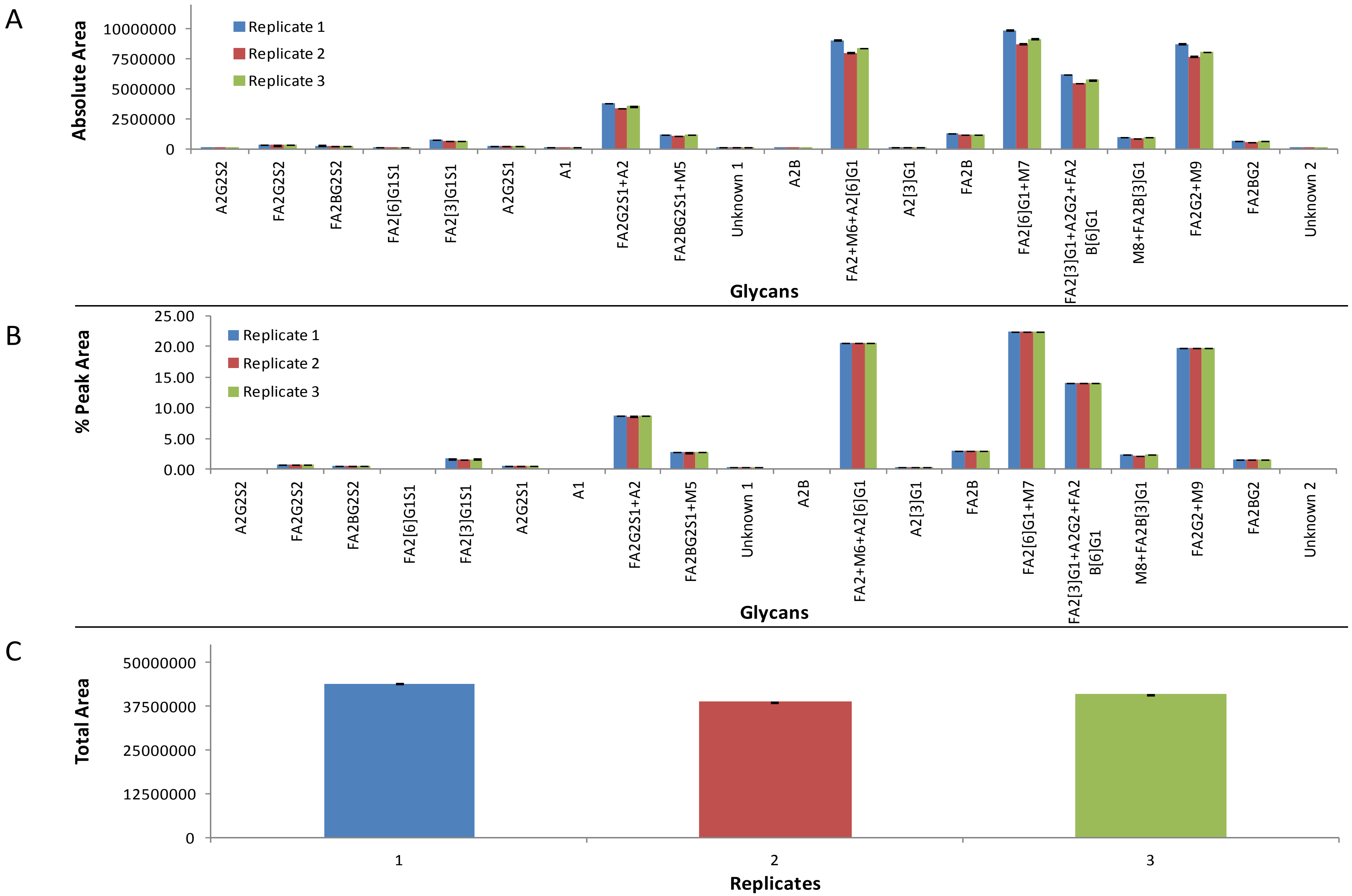

Figure S16. Statistical analysis of site S. Three chemical replicates were prepared; three injections were performed for each replicate. (A) Absolute peak area for each integrated peak. (B) Relative peak areas for each integrated peak. (C) Total peak area of each replicate.
